# Attrition in Conversational Agent–Delivered Mental Health Interventions: Systematic Review and Meta-Analysis

**DOI:** 10.2196/48168

**Published:** 2024-02-27

**Authors:** Ahmad Ishqi Jabir, Xiaowen Lin, Laura Martinengo, Gemma Sharp, Yin-Leng Theng, Lorainne Tudor Car

**Affiliations:** 1 Lee Kong Chian School of Medicine Nanyang Technological University Singapore Singapore Singapore; 2 Future Health Technologies Singapore-ETH Centre Campus for Research Excellence And Technological Enterprise Singapore Singapore; 3 Department of Neuroscience Monash University Melbourne Australia; 4 Centre for Healthy and Sustainable Cities Wee Kim Wee School of Communication and Information Nanyang Technological University Singapore Singapore Singapore; 5 Department of Primary Care and Public Health School of Public Health Imperial College London London United Kingdom

**Keywords:** conversational agent, chatbot, mental health, mHealth, attrition, dropout, mobile phone, artificial intelligence, AI, systematic review, meta-analysis, digital health interventions

## Abstract

**Background:**

Conversational agents (CAs) or chatbots are computer programs that mimic human conversation. They have the potential to improve access to mental health interventions through automated, scalable, and personalized delivery of psychotherapeutic content. However, digital health interventions, including those delivered by CAs, often have high attrition rates. Identifying the factors associated with attrition is critical to improving future clinical trials.

**Objective:**

This review aims to estimate the overall and differential rates of attrition in CA-delivered mental health interventions (CA interventions), evaluate the impact of study design and intervention-related aspects on attrition, and describe study design features aimed at reducing or mitigating study attrition.

**Methods:**

We searched PubMed, Embase (Ovid), PsycINFO (Ovid), Cochrane Central Register of Controlled Trials, and Web of Science, and conducted a gray literature search on Google Scholar in June 2022. We included randomized controlled trials that compared CA interventions against control groups and excluded studies that lasted for 1 session only and used Wizard of Oz interventions. We also assessed the risk of bias in the included studies using the Cochrane Risk of Bias Tool 2.0. Random-effects proportional meta-analysis was applied to calculate the pooled dropout rates in the intervention groups. Random-effects meta-analysis was used to compare the attrition rate in the intervention groups with that in the control groups. We used a narrative review to summarize the findings.

**Results:**

The systematic search retrieved 4566 records from peer-reviewed databases and citation searches, of which 41 (0.90%) randomized controlled trials met the inclusion criteria. The meta-analytic overall attrition rate in the intervention group was 21.84% (95% CI 16.74%-27.36%; I^2^=94%). Short-term studies that lasted ≤8 weeks showed a lower attrition rate (18.05%, 95% CI 9.91%- 27.76%; I^2^=94.6%) than long-term studies that lasted >8 weeks (26.59%, 95% CI 20.09%-33.63%; I^2^=93.89%). Intervention group participants were more likely to attrit than control group participants for short-term (log odds ratio 1.22, 95% CI 0.99-1.50; I^2^=21.89%) and long-term studies (log odds ratio 1.33, 95% CI 1.08-1.65; I^2^=49.43%). Intervention-related characteristics associated with higher attrition include stand-alone CA interventions without human support, not having a symptom tracker feature, no visual representation of the CA, and comparing CA interventions with waitlist controls. No participant-level factor reliably predicted attrition.

**Conclusions:**

Our results indicated that approximately one-fifth of the participants will drop out from CA interventions in short-term studies. High heterogeneities made it difficult to generalize the findings. Our results suggested that future CA interventions should adopt a blended design with human support, use symptom tracking, compare CA intervention groups against active controls rather than waitlist controls, and include a visual representation of the CA to reduce the attrition rate.

**Trial Registration:**

PROSPERO International Prospective Register of Systematic Reviews CRD42022341415; https://www.crd.york.ac.uk/prospero/display_record.php?ID=CRD42022341415

## Introduction

### Description of the Problem

Mental health disorders are among the largest contributors to the global disease burden, affecting 1 in every 8 people, or 970 million people around the world [1,2]. However, access to evidence-based interventions for the prevention and treatment of mental health disorders is limited [3,4]. This is due to various factors such as a lack of mental health services and professionals, poor mental health literacy, fear of stigma, and low perceived need for treatment [5-10]. There is a need for scalable and accessible mental health services. Digital technologies such as smartphones or websites are increasingly being used for the delivery of mental health interventions and have the potential to improve access to mental health care. Digital mental health interventions allow for the scalable delivery of diverse therapeutic approaches such as cognitive behavioral therapy and mindfulness for the treatment of mental health conditions such as depression, anxiety, substance abuse, and eating disorders [11-16].

### Description of the Intervention

Conversational agents (CAs) or chatbots are a more recent type of digital intervention, and they are becoming a popular method to deliver mental health interventions. CAs can be defined as computer algorithms designed to simulate human conversations textually or via speech through an interface [17]. CA-delivered mental health interventions (CA interventions) combine the delivery of psychotherapeutic content with an automated dialogue system that simulates the interaction between a mental health expert and the user [18]. These interventions provide an alternative avenue of psychotherapy to individuals who are not able to access mental health services owing to issues regarding time, location, or availability of resources [19]. CAs can also be a useful addition to traditional in-person therapy [20,21]. The presence of a CA can further contribute to improved therapeutic alliances with users to enhance adherence to the intervention [22,23]. Evidence for the efficacy of CAs in delivering mental health support is growing steadily. A recent meta-analysis showed that CA-delivered psychotherapy in adults significantly improved depressive symptoms with a medium effect size [19]. Providing self-guided therapy remotely via CAs may help address barriers to mental health access such as cost, long waiting time, and stigma [24]. Although the impact of mental health interventions delivered by CAs seems promising, studies evaluating such interventions also suggest high study attrition among participants [19]. Attrition or dropout occurs when participants do not complete the randomized controlled trial (RCT) assessments or complete the research protocol.

Digital health interventions typically report rapid and high attrition [13,25]. The overall attrition rate quantifies the level of attrition for the whole sample in a clinical trial, and the differential attrition rate refers to the level of attrition in the intervention group compared with that in the comparison group [26]. Attrition in clinical trials may introduce bias by modifying the random composition of the trial groups, limiting the generalizability of the study, and reducing the study power owing to reduced sample size [13,27]. To improve the quality of future clinical trials on CA interventions, there is a need to determine the attrition rates and the factors contributing to attrition in CA interventions.

### Why Is It Important to Conduct This Review?

There is scant evidence on the possible factors associated with attrition in CA interventions for mental health and health care in general. The review conducted by Lim et al [19] on the effectiveness of CA interventions for depression and anxiety symptoms indicated that almost a fifth of the participants (19%) attrited throughout the trials without exploring factors associated with the attrition. This was comparable with other reviews on digital health and digital mental health interventions reporting attrition rates that ranged from 24.1% to 47.8% after adjusting for publication bias [13,28]. In general, factors shown to be associated with attrition in trials of digital health interventions include poor user experience, a lack of perceived value, and privacy concerns [28,29]; for example, studies on mental health apps reported technical issues and errors that might affect users’ overall experience [15,30]. Qualitative findings further suggested that factors such as a lack of human interactions in digital health interventions and users’ technological competence also played a role in participants’ attrition [31].

In addition, for smartphone-based mental health interventions, providing monetary compensation and reminders to engage were associated with significantly lower attrition rates [13]. Conversely, participants in the intervention condition were more likely to drop out than the waitlist participants [13,32]. These reviews focused only on smartphone-delivered interventions and included studies published before 2020, omitting several more recently published studies on CA interventions. To fully harness CA interventions, there is a need to better understand the factors associated with both overall attrition as well as differential attrition in these interventions.

To this end, we aimed to (1) estimate the overall and differential rates of attrition in CA interventions, (2) evaluate the impact of the study on design- and intervention-related aspects on the overall and differential attrition rates in CA interventions, and (3) map and describe study design features aimed at reducing or mitigating study attrition in the trials.

## Methods

### Overview

We performed a systematic review of attrition rates in RCTs of CA health interventions in line with the Cochrane gold standard methodology [33] and the meta-analysis approach outlined by Linardon and Fuller-Tyszkiewicz [13]. We reported this review in line with the PRISMA (Preferred Reporting Items for Systematic Reviews and Meta-Analyses) guidelines [34]. The PRISMA checklist is included in Multimedia Appendix 1.

### Criteria for Study Selection

#### Types of Studies

Our inclusion criteria included RCTs, cluster RCTs, crossover RCTs, quasi-RCTs, and pilot RCTs in English. We decided to include these variations of RCTs because the field is still nascent, and findings from different forms of RCTs could be beneficial to understand the attrition rate in CA interventions. The publication types included peer-reviewed journals and conference papers that were published up to June 2022.

#### Types of Participants

Participants’ characteristics included healthy participants and participants with subclinical or clinically diagnosed mental health disorders such as depression, anxiety, attention-deficit/hyperactivity disorder, and bipolar disorder. Participants of any age were included so long as they personally interacted with the CA.

#### Types of Interventions

We included studies reporting a synchronous 2-way exchange with the participants via a CA. We excluded studies where the CA dialogues were wholly or partially delivered by human operators (*Wizard of Oz*) and studies with asynchronous response systems.

The interventions included either the delivery of psychotherapeutic content or those that provided training to improve mental well-being, reduced the symptoms of mental health conditions, or both. This included studies that aimed to reduce the symptoms of depression for clinical and subclinical populations or studies implementing mindfulness training for healthy adults. Detailed inclusion and exclusion criteria are outlined in Multimedia Appendix 2 [13,17,33,35-39].

#### Types of Outcome Measures

The primary outcome was the reported attrition number and the attrition rate calculated by the weighted risk of attrition of participants against the sample size of the studies for participants assigned to the CA intervention who then discontinued the study. This included the total attrition rate and the differential attrition rate in the intervention and comparison groups.

### Search Methods for the Identification of Studies

The search strategy included index terms and keywords that describe CAs, such as “conversational agent,” “embodied conversational agent,” “virtual assistant,” and “virtual coach” (Multimedia Appendix 3). The search strategy was previously developed for our scoping reviews [40,41] and was updated to include studies from 2020 to 2022 with the assistance of a medical librarian. We conducted the searches in the following databases on June 6, 2022: PubMed, Embase (Ovid), PsycINFO (Ovid), Cochrane Central Register of Controlled Trials, and Web of Science. In addition, we conducted a gray literature search on the first 200 entries from Google Scholar as suggested by the literature for the most optimal combination of databases [42,43]. We did not include any filter terms in the search. We also performed citation chasing by searching for all records citing ≥1 of the included studies (forward citation chasing) and all records referenced in the included studies (backward citation chasing) using *Citationchaser* [44]. The tables of excluded studies are presented in Multimedia Appendix 4.

### Data Collection and Analysis

#### Selection of Studies

On updating the searches from 2020 to 2022, we imported all identified references from the different electronic databases into a single file. The duplicated records were removed using *revtool* on R [35] and manually on Zotero (Corporation for Digital Scholarship). One reviewer (AIJ) performed the title and abstract screening using ASReview [36], an open-source machine learning software tool. The tool uses an active learning algorithm to actively sort and re-sort the records by prioritizing the most relevant records first based on the user’s inclusion and exclusion decisions. The title and abstract screening steps are detailed in Multimedia Appendix 2.

Three reviewers (AIJ, XL, and LM) were engaged in the full-text review. One reviewer (AIJ) retrieved the full text of the studies, and 2 reviewers (AIJ and XL) assessed their eligibility independently and in parallel. Any disagreements were discussed and resolved between the reviewers and with a third reviewer (LM) acting as the arbiter. Studies that were identified in our previous reviews (up to 2020) [41] and met the inclusion criteria of this review were included based on discussions among the 3 reviewers (AIJ, XL, and LM).

#### Data Extraction and Management

Data were extracted using a data extraction form on Microsoft Excel. The data extraction form was piloted by 2 reviewers (AIJ and XL) on the same papers (5/41, 12%) and amended in line with the feedback. We also included additional fields as required from the data extraction form that we referenced from Linardon and Fuller-Tyszkiewicz [13]. Four reviewers (AIJ, XL, GS, and Nileshni Fernando) extracted the data independently and in parallel.

We extracted the year of publication; study design; the type of comparison group (active or inactive); the type of intervention; and details of the CAs, including the type of CA (rule based or artificial intelligence [AI] enhanced), the personality of the CA [17], the level of human support, the presence of a reminder mechanism, and the input and output modalities of the CA. In addition, we extracted information on the study duration, compensation paid to study participants, and any other mechanism included specifically to increase user engagement. Any disagreements among the reviewers were resolved by discussion.

#### Assessment of the Risk of Bias in Included Studies

Four reviewers (AIJ, XL, GS, and Nileshni Fernando) independently assessed the risk of bias in the included studies using the Cochrane Risk of Bias Tool 2.0 [33] and visualized using *robvis* [45]. The risk of bias assessment was piloted with 10 (24%) of the 41 studies for consistency and clarity of judgment by 2 reviewers (AIJ and XL). The steps involved in the assessment of the risk of bias are detailed in Multimedia Appendix 2, and we have provided a summary, along with a table, in Multimedia Appendix 5. We requested clarification or more data from the authors of 1 (2%) of the 41 studies but did not receive any response even after sending a reminder 2 weeks later. The assessment of publication bias was reported via funnel plots and the Egger test for publication bias [37].

#### Data Analysis

The meta-analysis was conducted based on the approach outlined by Linardon and Fuller-Tyszkiewicz [13] and the Cochrane Handbook for Systematic Reviews of Interventions (version 6.3) [33]. We defined attrition as the number of participants who dropped out of the study during the intervention period by not completing the postintervention assessment. We did not include the follow-up period [13]. For crossover design studies, we only included the data before the crossover following the aforementioned definition. The second part of the crossover was not considered as the follow-up period.

The study’s overall attrition rate was estimated by calculating the weighted pooled event rate using random-effect models based on a meta-proportional approach [33] using Freeman-Tukey double arcsine transformed proportion [38]. This indicated the relative risk of attrition against the sample size of the studies for participants assigned to the CA intervention group. The aim of this overall attrition analysis was to compute the overall rate of attrition in the intervention group after controlling for the different sample sizes in the included studies. Event rates were then converted to percentages of events per 100 participants and calculated separately for all included studies (short-term studies [≤8 wk from baseline] as well as longer-term studies [>8 wk from baseline]). We used short-term and long-term groupings to facilitate a comparison between our results and those of the previous study on attrition in smartphone-delivered interventions for mental health problems [13].

The differential attrition rate was calculated as the odds ratio (OR) of the likelihood to attrit between the CA intervention condition and the comparison condition. The aim of the differential attrition analysis was to understand the odds of attrition compared with the control group. The ORs were calculated using random-effect models separately for short-term and long-term studies weighted by their inverse variance. Studies with 0 events in both arms were weighted as 0, and a correction of 0.5 was added to the arm with 0 events as a continuity correction. A log OR of >1 indicated a higher likelihood of attrition in the CA intervention groups compared with the control groups. We also conducted subgroup analyses to explore the sources of heterogeneity in both overall and differential meta-analyses. The detailed meta-analysis procedure and subgroup analyses conducted are specified in Multimedia Appendix 2. We also performed post hoc sensitivity analyses for subgroups with <5 studies because the estimate of tau-square might be imprecise [39]. In addition, we conducted exploratory analyses of all included studies regardless of the intervention duration using the same set of prespecified subgroup analyses on the overall and differential meta-analyses.

Meta-analysis was not conducted on the participant-level factors and the predictors of attrition owing to variability in reporting. We also identified additional factors significantly associated with attrition (*P*<.05) in the included RCTs. We collated and narratively presented these factors associated with attrition as reported by the studies.

## Results

### Overview

**Figure 1:**
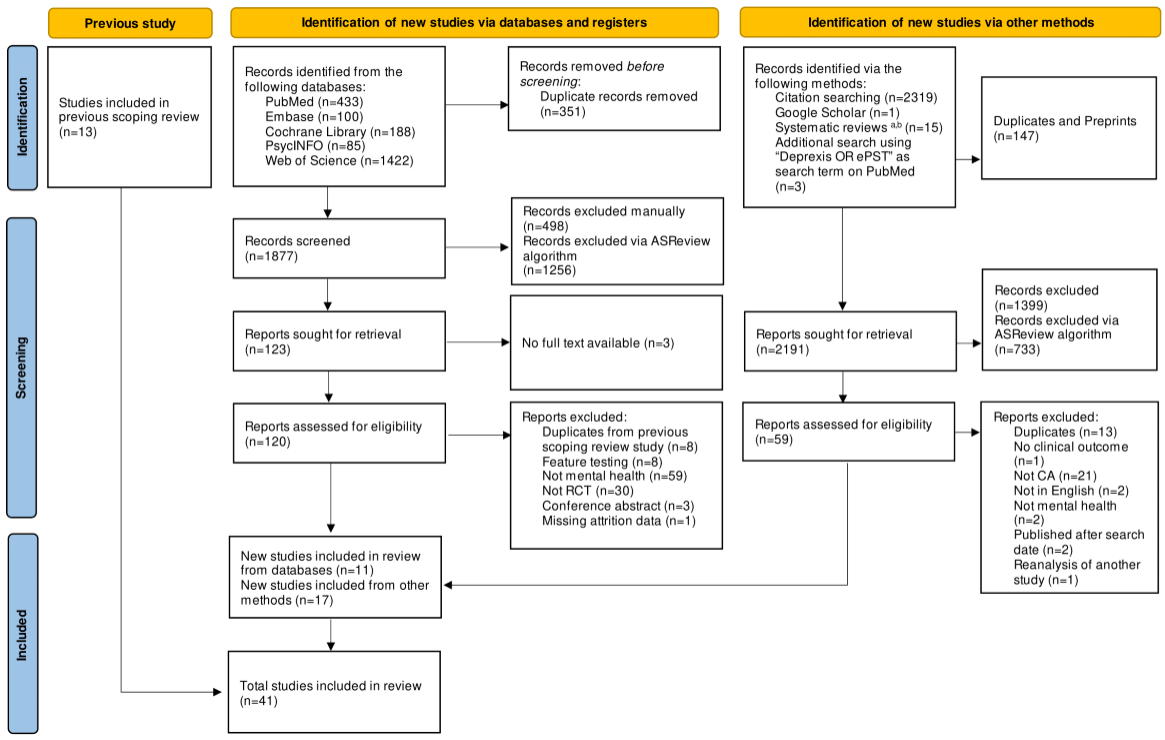
PRISMA (Preferred Reporting Items for Systematic Reviews and Meta-Analyses) flowchart. CA: conversational agent; ePST: electronic problem-solving treatment; RCT: randomized controlled trial. ^a^[19]; ^b^[46].

The updated search strategy retrieved 2228 records from peer-reviewed databases and 2319 from citation searching. After removing duplicates, of the total 4547 (2228+2319) records, 4030 (1877+2319[citation searching]-147[duplicates in citation searching]-19[records from other methods]) (88.63%) were screened on ASReview. Of these 4030 studies, 179 (4.4%) were then considered for full-text screening. We included 11 (6.1%) of the 179 studies identified from the full-text screening. We further identified 2 systematic reviews on CA intervention and included 14 studies that were not identified from our search strategy [19,46]. These studies used the *Deprexis* and electronic problem-solving treatment (ePST) interventions that did not explicitly identify themselves as CAs; for instance, both *Deprexis* and ePST described themselves as simulated dialogue that tailored their responses based on users’ input [47,48]. Subsequently, we followed up with an additional search on PubMed using “Deprexis OR ePST” as the search term and included 3 additional studies. We also included 13 studies identified in our previous review [41]. Thus, the total number of included studies is 41 (studies included in previous scoping review: n=13, 32%; new studies included from databases: n=11, 27%; and new studies included via other methods: n=17, 41%). Figure 1 presents the study selection process.

### Study Characteristics

Of the 41 studies included in this review, 22 (54%) were published before 2020 [15,47-67], and 19 (46%) were published in 2020 or later [14,68-85] (Table 1). Most of the studies were conducted in the United States (13/41, 32%) [14,15,48,56,58,59,64-66,68,70,82,85] and Germany (13/41, 32%) [47,50,52-55,60-62,69,75], with some studies (2/41, 5%) conducted in multiple countries such as Switzerland and Germany [51] and Switzerland, Germany, and Austria [57]. Most of the interventions were short-term interventions and lasted ≤8 weeks (26/41, 63%) [14,15,48,49,52,56,58,62-68,70-74,76-81,84], whereas some of the studies (15/41, 37%) lasted >8 weeks [47,50,51,53-55,57,59-61,69,75,82,83,85].

**Table 1:** Characteristics of included studies (n=41).

Study characteristics		Values, n (%)
**Year of publication**		
	Before 2020	22 (54)
	2020 or later	19 (46)
**Country**		
	United States	13 (32)
	Germany^a,b^	13 (32)
	South Korea	3 (7)
	Switzerland^a,b^	2 (5)
	United Kingdom	2 (5)
	Other (European Union)^b,c^	5 (12)
	Other^d^	6 (15)
**Type of study design**		
	RCT^e^	29 (71)
	Pilot RCT	8 (20)
	Crossover RCT	4 (10)
**Study duration**		
	≤8 wk	26 (63)
	>8 wk	15 (37)
**Sample population**		
	General population	11 (27)
	Population considered at risk	18 (44)
	Clinical population	12 (29)
**Target clinical outcome**		
	Treatment and monitoring	17 (41)
	Education and training	24 (59)
**Target disorder**		
	Depression only	17 (41)
	Mental well-being	9 (22)
	Co-occurring depression and anxiety	5 (12)
	Other^f^	10 (24)
**Type of control**		
	Waitlist control	18 (44)
	Active control	15 (37)
	Treatment as usual	8 (20)
**Enrollment method**		
	Remote enrollment option only	23 (56)
	In-person enrollment option provided	16 (39)
	Not specified	2 (5)
**Session type**		
	Defined session length	29 (71)
	User-determined session length	12 (29)
**Attrition range (%)**		
	0-10	13 (32)
	11-20	6 (15)
	21-30	11 (27)
	31-40	2 (5)
	41-50	3 (7)
	>50	6 (15)

^a^Conducted in both Switzerland and Germany.

^b^Conducted in Switzerland, Germany, and Austria.

^c^Ireland, Sweden, Italy, and the Netherlands.

^d^Japan, Ukraine, Argentina, New Zealand, China, and Russia.

^e^RCT: randomized controlled trial.

^f^Anxiety only, panic disorder, height phobia, gambling disorder, substance abuse, attention-deficit/hyperactivity disorder, irritable bowel syndrome, eating disorder, and personality disorder.

Psychoeducation and training were the focus of 24 (59%) of the 41 studies [47-57,59-62,64,66,69,70,75,77-79,84]. In almost half of the studies (18/41, 44%), participants were screened for mental health symptoms before the start of the study [14,50,52-56,59,62,63,66,68,72-74,80,82,83], and more than half of the studies (23/41, 56%) enrolled participants remotely using the web or the telephone [14,15,47,49-53,56,57,62,64,65,68-70,72,75,77,81-84]. More than one-third of the studies (17/41, 41%) focused on depression as the target disorder [47,48,50-56,58-62,67,69,83]. Of the 41 studies, 18 (44%) used waitlist control group participants [14,48-54,56,58,62,63,66,68,72,77,78,82], and 15 (37%) used an active control that included information or self-help (n=10, 67%) [15,60,65,71,74-76,80,81,83], alternative or comparable treatments such as stress-management cognitive behavioral therapy without a digital component (n=3, 20%) [73,84,86], or symptoms rating only (n=2, 13%) [64,70].

In the intervention group, of the 41 studies, 13 (32%) reported attrition between 0% and 10% [15,48,49,51,58,59,63,65,70,71,73,79,80], 6 (15%) reported attrition between 11% and 20% [14,57,67,74,78,83], 11 (27%) reported attrition between 21% and 30% [47,52,54-56,60,61,66,69,72,85], 2 (5%) reported attrition between 31% and 40% [53,64], 3 (7%) reported attrition between 41% and 50% [68,75,81], and 6 (15%) reported >50% attrition [50,62,76,77,82,84].

### Risk-of-Bias Assessment

**Figure 2:**
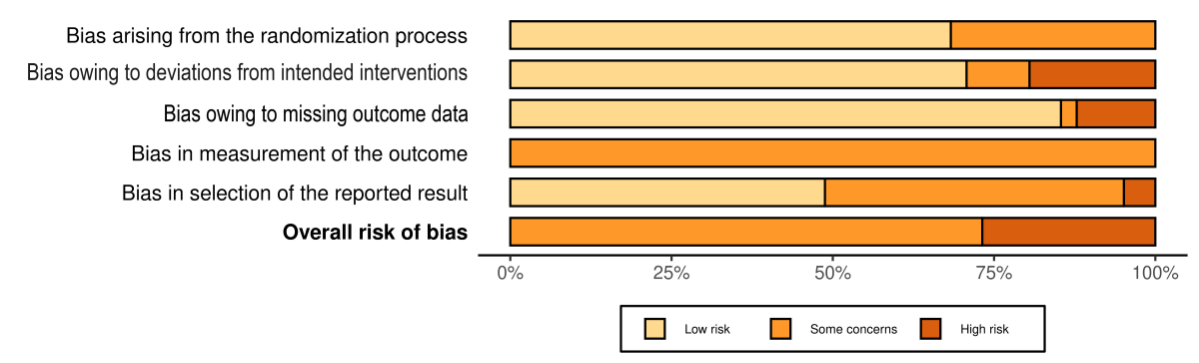
Summary plot for the risk-of-bias assessment of the included studies.

The most common risk of bias in the included studies was the bias in the measurement of the outcomes because they were all self-reported outcomes (Multimedia Appendix 5). The second most common bias was due to the selection of the reported results because most of the studies (18/41, 44%) [15,48-51,58,59,62,64,66,70,71,74,77,80,81,83,84] did not cite the RCT protocol or statistical analysis plan. Most of the studies (29/41, 71%) reported an intention-to-treat analysis. Figure 2 shows the summary plot of the risk of bias.

### CA Characteristics

Most of the CAs were rule based (29/41, 71%), and 12 (29%) were AI enhanced using natural language processing or other AI-based algorithms (Table 2). More than one-third of the studies (15/41, 37%) did not describe any specific visual representation of the CA. These were mainly studies that included *Deprexis* or *Deprexis-*based interventions (14/15, 93%) because they did not specifically identify themselves as CAs but used dialogue-based interventions. Of the 41 studies, 14 (34%) included an avatar or a static visual representation of the CA and 8 (20%) represented the CA using an embodied CA (ECA). With regard to the ECAs, of the 8 studies, 4 (50%) used relational agents, 3 (38%) used 3D-generated renders, and 2 (25%) used prerecorded videos. The CAs mostly presented a coach-like personality characterized by encouraging and nurturing personalities (19/41, 46%), followed by a factual personality characterized by being nonjudgmental and offering responses based on facts and observations (14/41, 34%). Of the 41 studies, in 5 (12%), the CA was designed to look like a physician or a health care professional, and in 3 (7%), the CA conversed with users using informal language characterized by exclamations, abbreviations, and emoticons in the dialogue (7%). More than one-third of the interventions were delivered via web-based applications (15/41, 37%), followed by those delivered by a stand-alone smartphone app (11/41, 27%).

**Table 2:** Characteristics of conversational agents (CAs; n=41).

Characteristics			Values, n (%)
**Type of** **CA**			
	No avatar or no visual representation	15 (37)	
	Avatar only	14 (34)	
	ECA^a^	8 (20)	
	Not specified	4 (10)	
**Delivery channel**			
	Web-based application	15 (37)	
	Stand-alone smartphone app	11 (27)	
	Computer- or laptop computer- or tablet computer–embedded program	7 (17)	
	Messaging app based^b^	7 (17)	
	Not specified	1 (2)	
**Dialogue modality**			
	Rule based	29 (71)	
	AI^c^ enhanced	12 (29)	
**Personality**			
	Coach like	19 (46)	
	Factual	14 (34)	
	Health care professional like	5 (12)	
	Informal	3 (7)	

^a^ECA: embodied CA.

^b^Slack, Facebook Messenger, or WeChat.

^c^AI: artificial intelligence.

### Study Attrition Rates

#### Overview

**Figure 3:**
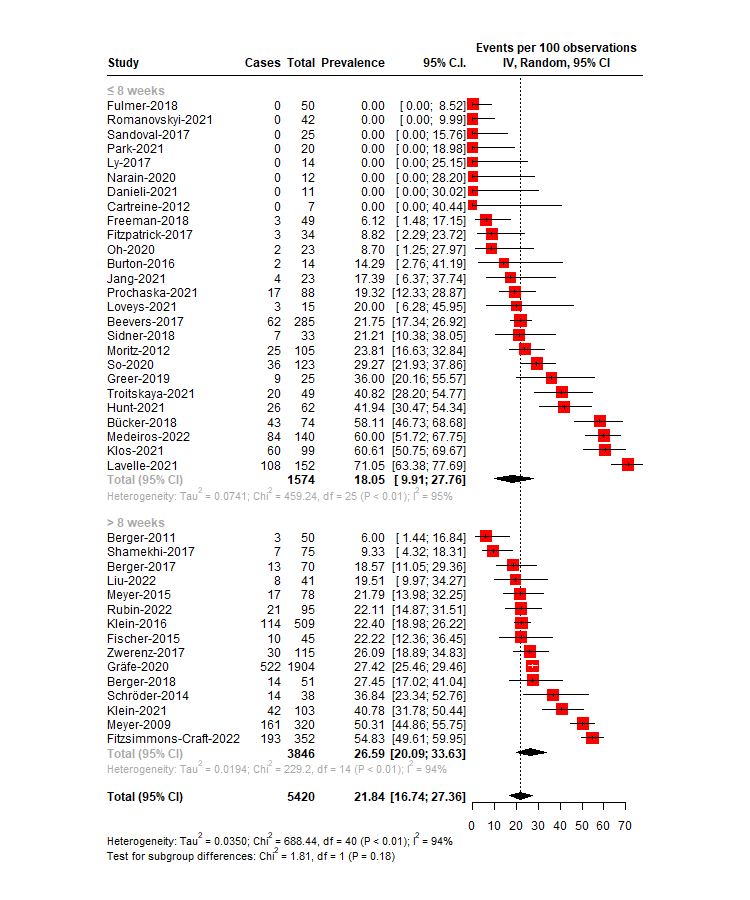
Overall attrition rates for the intervention group in conversational agent–delivered mental health interventions.

The overall weighted attrition rate for the intervention groups in all included studies was 21.84% (95% CI 16.74%-27.36%; *I*^2^=94%), whereas the differential attrition rate differed from 0% (log OR 1.28, 95% CI 1.10-1.48; *I*^2^=34.6%), indicating that the participants who received CA interventions were more likely to attrit than the control group participants. Figure 3 [14,15,47-85] shows the attrition rates for all included studies.

#### Short-Term Studies (≤8 Wk)

##### Overview

In the short-term studies, the overall weighted attrition rate in the intervention groups was 18.05% (95% CI 9.91%-27.76%), and there was evidence of high trial heterogeneity (*I*^2^=94.6%, 95% CI 93.05%-95.74%). The high heterogeneity was due to high variations among the studies in terms of symptoms, types of interventions, and study populations. Of the 26 studies, 5 (19%) reported 0% attrition in the intervention group [48,49,70,73,86]. The lowest attrition rate was 6.12% (95% CI 1.48%-17.15%) [63], and the highest was 71.05% (95% CI 63.38%-77.69%) [77].

The differential attrition rate did not differ from 0% (log OR 1.22, 95% CI 0.99-1.50), indicating that the attrition rates were similar across the intervention and control groups.

The heterogeneity was low to moderate (*I*^2^=21.89%, 95% CI 0%-54.6%). Of the 26 studies, 1 (4%) was identified as a potential outlier [15]. Removing this study from the model improved the *I*^2^ value greatly (*I*^2^=1.49%, 95% CI 0%-49.68%), and the differential attrition rate differed from 0% (log OR 1.27, 95% CI 1.04-1.54). This indicated that the attrition rate in the intervention group was larger than that in the control group after removing the outlying study. Multimedia Appendix 6 [14,15,47-85] shows the forest plot for the differential attrition meta-analysis [14,15,47-85].

##### Publication Bias for Short-Term Studies (≤8 Wk)

**Figure 4:**
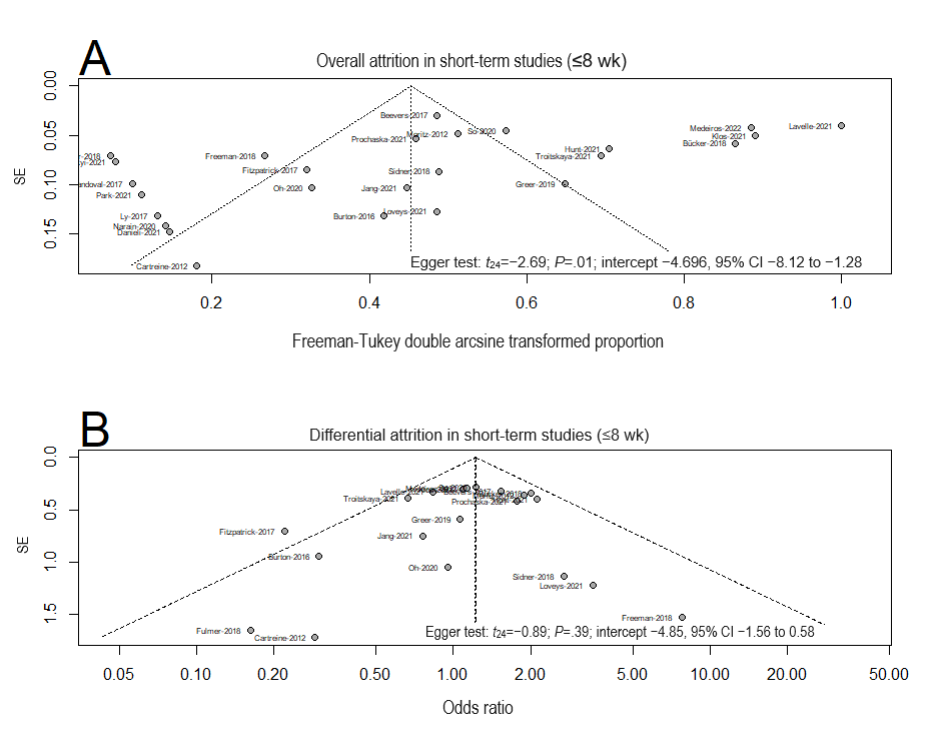
Funnel plots and the Egger test for publication bias for (A) overall attrition and (B) differential attrition in meta-analyses of the short-term studies.

For the overall attrition rate, the Egger test was significant (intercept −4.70, 95% CI −8.12 to −1.28; *P*=.01), indicating possible publication bias. A closer look at the funnel plot showed missing studies toward the bottom right of the plot, which suggested possible publication bias for small sample–sized studies with high attrition rates (Figure 4A [14,15,48,49,52,56,58,62-68,70-74,76-81,84]). For the differential attrition rate, the funnel plot indicated evidence of plot symmetry, and the Egger test was not significant (intercept −4.85, 95% CI −1.56 to 0.58; *P*=.39; Figure 4B [14,15,48,49,52,56,58,62-68,70-74,76-81,84]).

##### Subgroup Analyses of the Attrition Rates in Short-Term Studies (≤8 Wk)

Subgroup analyses were conducted for both overall attrition rate (Table 3) and differential attrition rate (Multimedia Appendix 6) in the CA-intervention groups compared with the control groups.

**Table 3:** Subgroup analyses for overall attrition rate in conversational agent (CA)–delivered mental health interventions.

Subgroups			Short-term studies (≤8 wk)										Long-term studies (>8 wk)							
			Interventions, n		Attrition rate, % (95% CI)		*I*^2^ (%)		*P* value			Interventions, n			Attrition rate, % (95% CI)		*I*^2^ (%)		*P* value	
**Risk of bias**										.21										.22
	High risk of bias	8		26.18 (15.69-38.13)		89.6					4			22.31 (17.85-27.10)		0				
	Low risk of bias	18		14.76 (4.27-29.25)		95.6					11			28.35 (20.25-37.20)		95.4				
**Funding source**										.71										.38
	Industry funding	9		20.43 (8.78-35.06)		92.4					4			32.36 (18.14-48.46)		97.0				
	Public funding only	17		16.56 (6.35-29.79)		95.1					11			23.19 (15.92-33.65)		92.1				
**Duration (wk)**										.43										.68
	0-4	17		15.57 (5.10-29.92)		95.3					N/A^a^			N/A		N/A				
	5-8	9		23.15 (11.13-37.67)		93.4					N/A			N/A		N/A				
	9-12	N/A		N/A		N/A					11			25.30 (19.35-31.75)		90.3				
	>13	N/A		N/A		N/A					4			30.04 (10.75-53.88)		96.1				
**Study design**										.65										.19
	RCT^b^	19		19.68 (10.34-30.95)		95.2					14			26.00 (19.33-33.27)		94.3				
	Pilot RCT	7		12.73 (0-38.45)		91.8					1			N/A		N/A				
**Type of disorder**										.80										.32
	Depression	6		17.89 (5.44-34.60)		91.5					11			24.03 (17.74-30.93)		91.4				
	Depression and anxiety	5		7.68 (0-40.93)		97.1					N/A			N/A		N/A				
	Mental well-being	9		24.29 (8.37-44.57)		94.2					N/A			N/A		N/A				
	Other^c^	4		20.02 (10.61-31.36)		81.9					3			33.77 (17.01- 52.90)		94.9				
**With CBT^d^**										.41										.65
	Yes	17		21.35 (11.26-33.41)		95.1					12			27.51 (20.16-35.31)		94.5				
	No	9		11.64 (0.21-33.29)		93.8					3			22.99 (7.94-42.72)		92				
**With mindfulness component**										.02										.27
	Yes	12		30.24 (17.02-45.27)		95.5					11			23.89 (17.67-30.71)		91.5				
	No	14		8.66 (0.89-21.20)		92.8					4			34.35 (18.00-52.84)		93.8				
**Personalization**										.63										.53
	No personalization	6		17.04 (5.08-33.33)		84.7					2			38.10 (10.38-70.94)		97.1				
	Minimal personalization	2		44.57 (2.50-92.50)		96.0					N/A			N/A		N/A				
	Substantial personalization	9		21.56 (12.52-32.12)		89.0					12			25.25 (19.16-31.85)		91.3				
	Major personalization	9		10.98 (0-35.05)		96.5					1			19.51 (8.61-33.23)		N/A				
**CA algorithm**										.42										.49
	Rule based	16		21.53 (11.72-33.12)		93.1					13			25.02 (19.30-31.19)		90.6				
	AI^e^ enhanced	10		13.25 (1.29-32.79)		96.2					2			37.16 (8.05-72.63)		95				
**Type of CA**										.14										.16
	No avatar	3		33.36 (15.39-54.24)		94.36					12			29.21 (21.81-37.19)		94.6				
	ECA^f^	6		8.03 (1.37-18.04)		59					2			15.35 (5.05-29.63)		80.3				
	Avatar	13		20.13 (6.67-37.97)		96.4					1			19.51 (8.61-33.23)		N/A				
	Not specified	4		15.15 (1.79-35.93)		86.4					N/A			N/A		N/A				
**With rewards component**										.74										<.001^g^
	Yes	15		16.63 (9.93-27.41)		92.4					1			54.83 (49.60-60.00)		89.9				
	No	11		20.07 (5.77;-39.39)		96					14			24.68 (19.20-30.59)						
**Reminder**										.20										.98
	With reminder	14		23.96 (12.19-37.99)		94.8					7			26.42 (14.80-39.96)		95.7				
	Without reminder	12		11.43 (1.76-26.27)		94.7					8			26.51 (17.85-36.16)		92.1				
**Delivery channel**										.02^g^										<.001^g^
	Web based	4		30.74 (15.26-48.70)		91.5					11			26.96 (20.73-33.66)		90.8				
	Computer- or laptop computer– or tablet computer–embedded program	6		4.72 (0.06-13.49)		61.5					1			9.33 (3.62-17.12)		N/A				
	Smartphone app	10		14.32 (5.71-25.56)		87.5					1			54.83 (49.60-60.00)		N/A				
	Messenging app based (meaning Slack, Facebook Messenger, or WeChat based)	6		33.27 (10.58-60.80)		96.9					1			19.51 (8.61-33.23)		N/A				
	Not specified	N/A		N/A		N/A					1			22.11 (14.27-31.06)		N/A				
**With blended component**										.38										.02
	Yes	3		19.12 (10.38-29.56)		95.1					6			18.65 (12.86-25.20)		75.4				
	No	23		9.89 (0.35-26.31)		38.8					9			32.54 (23.01-42.84)		94.9				
**Enrollment method**										.15										.12
	Remote options only	14		26.63 (15.19-39.82)		95.4					9			30.18 (20.38-40.95)		95.6				
	With in-person option	10		9.68 (0.15-27.14)		92					6			21.28 (16.69-26.26)		53.7				
	Not specified	2		5.02 (0-33.08)		88					N/A			N/A		N/A				
**Study population**										.05^g^										.41
	At risk	11		19.92 (1.53-29.81)		90.1					7			30.10 (17.09-44.93)		96.4				
	Clinical	4		3.53 (0-13.05)		36					8			23.87 (18.55-29.61)		76.5				
	General	11		22.05 (6.73-42.30)		96.1					N/A			N/A		N/A				
**Session length**										.61										.34
	Defined session length	15		20.26 (9.93-32.79)		94.1					14			27.05 (20.28-34.38)		94.3				
	User determined	11		15.31 (3.17-33.12)		95.4					1			19.51 (8.61-33.23)		N/A				
**With symptom trackers component**										.99										.003
	Yes	14		17.77 (6.44-32.52)		93.9					6			16.36 (10.32-23.39)		64.1				
	No	12		18.22 (7.21-32.44)		95.3					9			33.48 (24.91-42.62)		95.4				

^a^N/A: not applicable.

^b^RCT: randomized controlled trial.

^c^Anxiety only, panic disorder, height phobia, gambling disorder, substance abuse, attention-deficit/hyperactivity disorder, irritable bowel syndrome, eating disorder, and personality disorder.

^d^CBT: cognitive behavioral therapy.

^e^AI: artificial intelligence.

^f^ECA: embodied CA.

^g^Subgroup analyses were not significant after dropping subgroups with <5 studies.

For the overall attrition rate, there were significant differences in the attrition rates in short-term studies depending on the inclusion of mindfulness content (*χ*^2^_1_=5.1; *P*=.02). Interventions that included mindfulness content (n=12) showed a higher rate of attrition in the intervention group (30.24%, 95% CI 17.02%-42.27%) compared with interventions without mindfulness content (n=14; 8.66%, 95% CI 0.89%-21.2%). There were also significant differences depending on the population types, delivery channels, and types of disorders. However, these differences were not significant after excluding subgroups with <5 studies.

Subgroup analysis of the differential attrition rates showed that there were significantly different attrition rates in the intervention group compared with the control group depending on the subdurations (*χ*^2^_1_=5.8; *P=*.02). There were also significantly different attrition rates between study populations (*χ*^2^_2_=9.3; *P*=.009), and the types of controls (*χ*^2^_1_=4.7; *P*=.03). The relative risks of attrition for studies that lasted between 5 and 8 weeks were significantly different (n=9; log OR 1.61, 95% CI 1.22-2.13) compared with studies that lasted <5 weeks (n=17; log OR 0.99, 95% CI 0.75-1.31). Studies that recruited populations considered to be at risk (n=9) showed significantly higher attrition rates in the intervention group than in the control group (log OR 1.65, 95% CI 1.26-2.15) when compared with general populations (n=7; log OR 0.96, 95% CI 0.71-1.30) and clinical populations (n=3; log OR 0.47, 95% CI 0.13-1.66). The subgroup analysis was still significant when compared between the general population and the group considered to be at risk only (*χ*^2^_1_=6.9; *P*=.03). Finally, there were higher attrition rates in the intervention studies than in studies that used waitlist controls (n=11; log OR 1.52 95% CI 1.18-1.95) than those that used active controls (n=7; log OR 0.96, 95% CI 0.69-1.54). Only 1 (2%) of the 41 studies used treatment as usual as the control group [67]. No other comparisons were significant.

#### Long-Term Studies (>8 Wk)

##### Overview

The weighted attrition rate for the intervention group in long-term studies was 26.59% (95% CI 20.09%-33.63%), and there was evidence of high trial heterogeneity (*I*^2^=93.89%, 95% CI 91.43%-95.64%). The lowest relative attrition rate was 6% (95% CI 1.44%-16.84%) [51], and the highest was 54.83% (95% CI 49.61%-59.95%) [77].

The differential attrition rate differed from 0% (log OR 1.33, 95% CI 1.08-1.65), indicating that the attrition rates in the intervention group were higher than those in the control group. The heterogeneity was moderate (*I*^2^=49.43%, 95% CI 0.083%-72.11%). However, 1 (2%) of the 41 studies was identified as a potential outlier [50]. Removing this study from the model improved the *I*^2^ value greatly (*I*^2^=24.22%, 95% CI 0%-59.80%), and the differential attrition rate still differed from 0% (log OR 1.22, 95% CI 1.05-1.42); again, this indicated that the attrition rates in the intervention group were significantly larger than those in the control group even after removing the outlying study. The outlying study [50] used a weighted randomization method in which 80% of the participants were allocated to the intervention group. The subgroup analyses were conducted without the outlier because the study seemed to explain >20% of the heterogeneity in the model. However, sensitivity analyses conducted with and without the outlying study did not change the results of the subgroup analysis.

##### Publication Bias in Long-Term Studies (>8 Wk)

**Figure 5:**
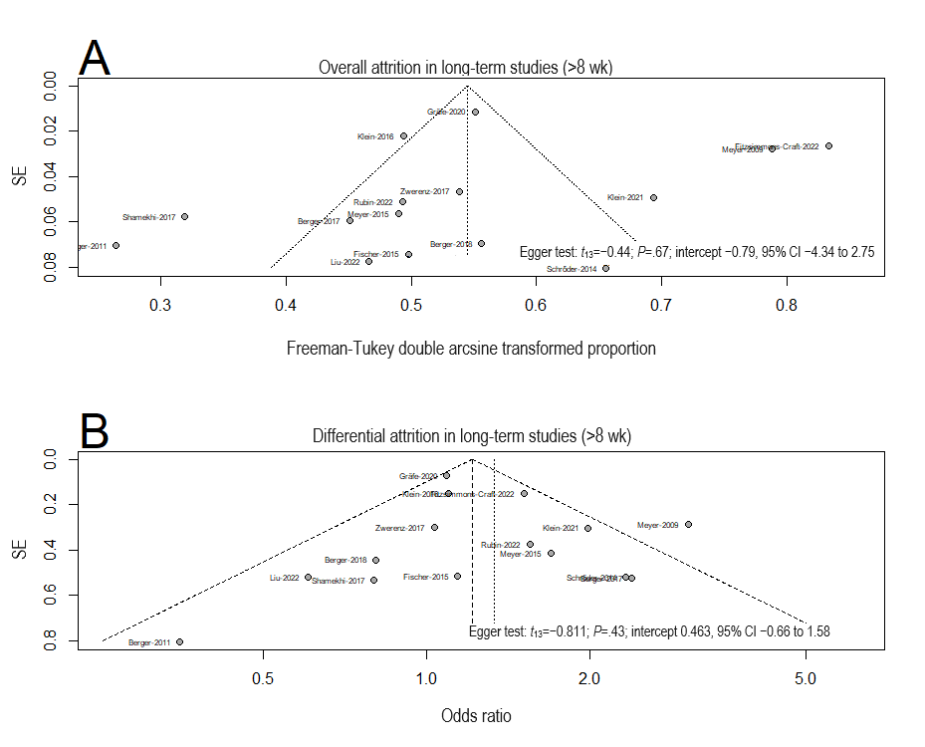
Funnel plots and the Egger test for publication bias for (A) overall attrition and (B) differential attrition in meta-analyses of the long-term studies.

For the overall attrition rate, the funnel plot indicated evidence of plot asymmetry, but the Egger test was not significant (intercept −0.79, 95% CI −4.34 to 2.75; *P*=.67), suggesting a low likelihood of publication bias. For the differential attrition rate, the funnel plot indicated evidence of plot symmetry, and the Egger test was not significant (intercept 0.46, 95% CI −0.66 to 1.58; *P*=.43; Figure 5 [14,15,47-85]).

##### Subgroup Analyses of the Attrition Rates in Long-Term Studies (>8 Wk)

For overall attrition, there were significant differences in the attrition rates in the intervention groups of long-term studies that had a blended design (*χ*^2^_1_=4.7; *P*=.03) and included symptom trackers or mood monitoring (*χ*^2^_1_=9.0; *P*=.003). Interventions that included blended designs (n=6) showed a significantly lower attrition rates (20.41%, 95% CI 15.49%-25.81%) than those without (n=9; 33.3%, 95% CI 23.01%-44.44%). Interventions that included symptom trackers (n=6) showed a significantly lower attrition rates (16.36%, 95% CI 10.32%-23.39%) than those without (n=9; 33.48%, 95% CI 24.91%-42.62%; Table 3). No other comparisons were significant.

The differential attrition rates were significantly different across studies that included mindfulness content compared with those without (*χ*^2^_1_=5.0; *P*=.03) and studies that targeted depression symptoms compared with those that targeted other mental health symptoms (*χ*^2^_1_=8.6; *P*=.003). Studies without mindfulness intervention showed higher attrition rates in the intervention groups than in the controls (n=10; log OR 1.56, 95% CI 1.25%-1.96%) compared with studies that included mindfulness content (n=4; log OR 1.11, 95% CI 1.05%-1.42%). Studies that targeted depression symptoms specifically showed relatively similar attrition rates in both intervention and control groups (n=10; log OR 1.09, 95% CI 0.96%-1.22%) compared with studies that targeted other mental health symptoms such as gambling disorder and substance abuse (n=4; log OR 1.63, 95% CI 1.28%-2.08%). No other comparisons were significant.

### Exploratory Subgroup Analyses

The overall weighted attrition rate for the intervention group in all included studies was 21.84% (95% CI 16.74%-27.36%; *I*^2^=94%). Exploratory subgroup analyses using the prespecified subgroup analyses were conducted to explain the heterogeneity of the included studies regardless of the duration of the interventions. We have included the full findings in Multimedia Appendix 7 and only highlight findings that differed from our prespecified analysis here. For overall attrition, as in our prespecified analysis, there were significant differences in the attrition rates in the intervention groups depending on the inclusion of mindfulness content (*χ*^2^_1_=4.0; *P*=.05). However, we did not find significant differences in the inclusion of blended support compared with nonblended intervention and the inclusion of symptom tracker compared with intervention without symptom tracker. In addition, we found significant differences depending on the type of CA used (*χ*^2^_3_=13.1; *P*=.005), the CA delivery channel (*χ*^2^_4_=21.3; *P*<.001), and the study enrollment method (*χ*^2^_2_=7.4; *P*=.02). Studies that did not use any identifiable avatar reported the highest rate of attrition (n=15; 30%, 95% CI 23.44%-37.01%), followed by studies that did not specify the use of an avatar or a visual representation of the CA (n=14; 20.12%, 95% CI 7.29%-36.82%), studies that used a static avatar (n=4; 15.15%, 95% CI 1.79%-35.93%), and studies that used an ECA (n=8; 10.3%, 95% CI 4.29%-18.04%). Interventions that were delivered via messaging app (meaning “Slack, Facebook Messenger, or WeChat” based) showed the highest rate of attrition (n=7; 31.19%, 95% CI 10.68%-56.28%), followed by those delivered by web-based applications (n=15; 27.9%, 95% CI 22.35%-33.78%), and those delivered by stand-alone smartphone apps (n=11; 17.36%, 95% CI 6.54%-31.48%). CAs installed on a computer, a laptop computer, or a tablet computer showed the lowest rate of attrition (n=7; 5.61%, 95% CI 1.09%-12.3%). Finally, studies that offered remote onboarding only (n=23) showed a higher attrition rate (28.42%, 95% CI 21.30%-36.1%) than studies that offered an in-person onboarding process (n=16; 15.01%, 95% CI 8.46%-22.82%).

The differential attrition rate differed from 0% (log OR 1.28, 95% CI 1.10-1.48; *I*^2^=34.6%), indicating that the participants who received CA interventions were more likely to attrit than the control group participants.

For differential attrition, our findings mostly concurred with our prespecified analysis. Unlike in the prespecified analysis, there was a significant difference for studies that included symptom trackers (*χ*^2^_1_=5.0; *P*=.02). Studies that included symptom trackers (n=17) showed relatively lower attrition in the intervention group than in the control group (log OR 1.02, 95% CI 0.81-1.29) compared with studies without symptom trackers (n=18; log OR 1.44, 95% CI 1.19-1.74).

### Additional Factors Associated With Attrition

Of the 41 studies, 16 (39%) assessed the association of different study features on participants’ attrition (short-term studies: n=8, 50%; long-term studies: n=8, 50%). We grouped the findings into two categories: (1) demographic predictors and (2) baseline measurement predictors or symptom severity.

The associations between participants’ demographics and attrition were assessed and reported in 10 (63%) of the 16 studies (short-term studies: n=4, 40%; long-term studies: n=6, 60%). Only 1 (25%) of the 4 short-term studies found age to be significantly associated with attrition. Participants who dropped out were found to be significantly younger than those who completed the whole intervention [62]. Other demographics-related factors assessed in these 10 studies were not associated with attrition, including sex, years of education, marital status, employment, actively receiving therapy or medication, and current diagnosis, for both short-term and long-term studies.

Of the 41 studies, 12 (29%) explored the association between baseline predictors or symptom severity and attrition (short-term studies: n=6, 50%; long-term studies: n=6, 50%). More severe baseline symptoms were associated with attrition for some of the short-term studies (2/6, 33%) but not for the long-term studies. Higher anxiety-related symptoms measured using the General Anxiety Disorder-7 questionnaire [62] and the Visceral Sensitivity Index [68] were significantly related to attrition. Other factors found to be associated with higher attrition included lower quality of life measured using the World Health Organization Quality of Life Brief Questionnaire [52], higher Fear of Food Questionnaire score [68], higher severity of gambling pathology measured using the Pathological Gambling Adaptation of the Yale-Brown Obsessive-Compulsive Scale [62], and lower self-esteem [81]. Of the 10 studies, 1 (10%) reported that participants who attrited significantly greater positive affect compared to those who completed the study using the Positive and Negative Affect Schedule [77].

## Discussion

### Principal Findings

To our knowledge, this systematic review and meta-analysis is the first to examine attrition in RCTs of CA interventions for mental health. A total of 41 RCTs met the inclusion criteria. Our findings showed that approximately one-fifth of the participants (18.05%) dropped out in short-term studies, and approximately a quarter (26.59%) dropped out in long-term studies. Participants who received CA interventions were more likely to attrit than the control group participants for both long-term and short-term studies. Several study-level moderators were identified. For short-term studies, higher overall attrition rates were found in intervention groups that included mindfulness content and those that included participants from the general population. Compared with the control group participants, participants in the short-term CA interventions were also more likely to attrit in studies that lasted >1 month, those that included participants considered to be at risk, and studies in which intervention group participants were compared against waitlist controls as opposed to alternative active controls. For long-term studies, higher overall attrition rates were found in interventions that did not include human support and studies that did not include a symptom tracker. Exploratory subgroup analyses conducted on all included studies regardless of the study duration largely supported the analysis except for the inclusion of blended support. In addition, exploratory analyses found that studies that used an ECA, delivered via a computer or smartphones, and provided in-person enrollment options were associated with lower attrition rates.

### Comparison With Prior Research

#### Overall Attrition

The overall attrition rates for short-term and long-term studies in our review are lower than the attrition estimates of short-term and long-term studies on smartphone-delivered interventions (24.1% and 35.5%, respectively) [13] and electronic mental health treatments for eating disorders (21%) [87]. Our findings are comparable with attrition rates in studies evaluating face-to-face mental health treatments such as interpersonal psychotherapy (20.6%) [88], individual psychotherapy for depressive disorders (19.9%) [89], and generalized anxiety disorders (17%) [90]. When focusing only on studies evaluating smartphone-delivered CAs in our review (n=11), we found that only 14.32% of the participants dropped out of the short-term studies and 17.36% dropped out of all included studies; these rates are lower than the estimated attrition rate previously reported for smartphone-delivered mental health interventions [13]. Taken together, the delivery channel may indirectly influence study attrition [13]. Although we found lower attrition in studies that were delivered via programs installed on a computer or a laptop computer compared with other delivery channels, these studies [48,58,59,63,79] were conducted in a laboratory setting compared with the participants’ environment, which might influence the retention rate.

#### Factors Associated With Attrition

CA interventions used as adjuvants of psychotherapy sessions or with human support may aid in retaining participants, particularly in long-term studies. A closer look at the long-term studies that included human support revealed that most of these studies (6/15, 40%) used the CA interventions as an adjunctive tool with no specific instruction given to the primary therapist on how to support participants’ journey through the CA interventions. This suggests that the presence of the therapist alone could suffice to retain participation in the study over a longer period. Although participants may be staying for the primary therapist and not engaging with the CA intervention, a study with both therapist-guided and unguided groups found no significant differences in the time spent with the CA intervention [51]. It is also possible that participants may have consulted their primary therapist about the CA interventions, which was not reported by the studies. A recent scoping review reported that many studies that included human support did not consistently report the type of support provided by humans [91]. This was similarly observed in our study because none of the included studies mentioned whether the therapist discussed the CA intervention during the participants’ routine sessions. Furthermore, it is also possible that participants within clinical settings are more likely to stay with the intervention, as was found in our results, unrelated to the blended support provided. Therefore, it is difficult to draw conclusions regarding the impact of using CA interventions adjunctively in terms of retaining participants within the study. However, this finding should be taken with caution because we found that there were no significant differences in the attrition rates between studies that included blended and nonblended approaches when we included all studies regardless of the study duration. This may be because fewer studies (9/41, 22%) included the blended approach overall.

In terms of the intervention content, short-term studies that included mindfulness content had higher overall attrition rates than those without mindfulness content. This was contrary to 2 previous meta-analyses that showed that the inclusion of the mindfulness component had no impact on attrition rates [13,28]. However, the attrition rate was similar to that in a systematic review of self-guided mindfulness interventions that reported an unweighted mean attrition rate of 31% (range 0%-79%) [92]. Future interventions may need to pay closer attention to participants’ engagement when including mindfulness content as part of a CA intervention. Factors such as using symptom trackers and personalized feedback may increase the engagement rate [93]. This is aligned with our findings and prior meta-analyses that suggest that including feedback may improve the retention rate [28].

Interestingly, our results also found relatively lower differential attrition rates in the intervention groups of long-term studies that included mindfulness compared with studies without. However, this finding was not replicated when we analyzed all studies regardless of the study duration. A recent review suggested that longer mindfulness practice sessions may be associated with the development of mindfulness skills [92]. Therefore, this result should be interpreted with caution because the relationship between the frequency and the duration of mindfulness practice sessions is still unclear [92].

Our study also found that including any form of visual representation of a CA may be associated with lower attrition rates compared with no visualization at all. This is aligned with many studies on the design of CAs that stressed the importance of design to create positive perceptions of the CA [94]. However, a recent scoping review reported that visual representation of the CA showed mixed and no association with subjective user experience [95]. A closer look at the studies included in this review suggested that most of them (35/41, 85%) lasted only 1 session [95]. It is possible that not having any CA visualization could affect user experience over time as alluded to by our findings. Future studies should explore the relationship between CA visual representation and user experience and study adherence over a longer duration.

In terms of the study sample population, sample populations considered to be at risk were more likely to attrit than samples from general and clinical sample populations. However, other meta-analyses of digital interventions for mental health issues found no difference in attrition rates across sample populations [13,28]. This finding is difficult to interpret because there could be multiple factors that may affect this relationship, such as symptom severity and other demographic factors not included in our analysis. Future studies may explore this relationship further to better understand this association.

#### Factors Not Associated With Attrition

Providing monetary incentives did not affect the attrition rates significantly. This finding is similar to that of a previous meta-analysis focusing on smartphone apps specifically for depressive symptoms [28] but contrary to that of a study focusing on smartphone interventions for mental health in general [13]. Time spent within the study may be a greater driver for attrition, as can be seen in the higher attrition rates for longer-term studies found in our results. However, research on the impact of monetary incentives on participants’ retention in digital health interventions is still in its infancy [96]. More needs to be done to understand how monetary incentives affect participants’ retention as well as effective engagement in the intervention.

Finally, several features such as providing reminders and the level of personalization provided by the CA also did not affect attrition rates. This is noteworthy because a personalized intervention that is responsive to users’ inputs is related to better engagement with the intervention [93]. There may be further nuances between attrition and effective engagement; for instance, factors that lower the risk of attrition might not be directly related to factors that promote study adherence [28].

### Strengths and Limitations

We conducted a comprehensive literature search that included both peer-reviewed databases and gray literature sources; in addition, we conducted backward and forward citation searches. As this is a nascent area, we prioritized the sensitivity of our search to capture the various representations of CAs.

However, our study has some limitations. First, some unpublished literature presented at niche conferences and meetings may have been omitted. In addition, some studies might have escaped our search strategy, as evidenced by the inclusion of *Deprexis*, *Deprexis-*based interventions, and ePST intervention that did not explicitly mention terms related to *conversational agent* in the studies concerned. Second, the heterogeneity of the mental health conditions addressed by the CA intervention made it difficult to generalize the findings to a specific disorder. The recommendations provided here should be taken as a general suggestion to improve retention rates in CA interventions for mental health but not for a specific mental health disorder. Third, our results indicated possible publication bias in short-term studies. A closer look at the funnel plot suggested a lack of small sample–sized studies (n<20) with high attrition rates in the intervention groups. It is possible that these studies were not being published because they could be too experimental and small scaled. However, it is possible that the findings are reported elsewhere at niche conferences and internal sharing, which may have been omitted based on our search strategy. Fourth, our results may not directly translate into understanding factors that may increase engagement with the interventions. Although we recognize that engagement is interlinked with attrition [97], the lack of consensus and reporting of engagement metrics limits our understanding of this relationship. A recent review identified several patient-, intervention-, and system-level factors associated with engagement [98]. However, many of these associations were not consistent across different digital mental health interventions, and there was poor consistency in the reporting of the metrics. We echoed others’ recommendations to include and standardize the reporting of engagement metrics to better understand the indicators of nonuse attrition [41,93,97,98]. Our findings can inform future researchers of the potential factors for attrition in CA interventions. These may serve as a basis to make informed decisions on the sample size required or to conduct further studies on the specific mechanisms that may or may not motivate attrition. Fifth and last, our subgroup analyses for all studies regardless of the intervention duration were exploratory post hoc analyses and should be interpreted as such.

### Implications and Recommendations

Several implications and recommendations emerged from our findings. First, researchers may want to account for the attrition of approximately one-third of the participants when designing RCTs involving CA interventions. This number may need to be further adjusted depending on the sample population, delivery modes, and comparison group used in the intervention to minimize the potential threats to the external and internal validity of studies that evaluate the efficacy of CA interventions for mental health. Second, researchers may want to consider including active controls in RCTs. Our results and the findings from other similar reviews on attrition in digital health research [13,28] suggested that comparing digital interventions with waitlist controls might not be the ideal way because participants in the comparison group may be more motivated to remain in the study than those in the intervention group [13]. Control interventions consisting of periodic mood assessments via an app or a nonconversational version of the app may be more appropriate for the assessment of CA effectiveness. Third, the inclusion of a visual representation of the CA may help create a more positive perception of the CA and reduce attrition. A recent review suggested that design considerations such as having a humanlike representation and having medical attire for the CA may be helpful to reduce attrition [95]. Fourth, CA interventions should be adjunctive to ongoing therapy sessions. Although the association between attrition and the use of blended support may be inconclusive, the use of CA interventions may further enrich participants’ experience between sessions and provide ongoing support to practice the skills learned during regular sessions. Fifth and last, clinicians interested in implementing CA interventions in their practice should be aware of the high attrition rate and should closely monitor patients’ progress within their practice.

### Conclusions

According to our findings, at least one-fifth of the intervention group participants in RCTs of CA interventions will drop out of the trials within the intervention period. This attrition rate is comparable with that of face-to-face mental health interventions and less than that of other digital health interventions. Furthermore, intervention group participants were more likely to drop out than control group participants. Attrition was lower in shorter-term studies, studies that included participants considered to be at risk, and studies in which intervention group participants were compared against waitlist controls as opposed to alternative active controls. In addition, not including mindfulness content or symptom trackers was found to be associated with a smaller risk of attrition. Future studies may benefit from delivering CA interventions in a blended setting, with symptom screening; comparing the CA interventions against active controls such as symptom tracking only without the CA component; and including a visual representation of the CA to reduce attrition rates in the intervention group.

## Appendix

Multimedia Appendix 1 PRISMA (Preferred Reporting Items for Systematic Reviews and Meta-Analyses) 2020 checklist.

Multimedia Appendix 2 Criteria for study selection and extended meta-analysis method.

Multimedia Appendix 3 Search strategy for PubMed.

Multimedia Appendix 4 Tables of excluded studies.

Multimedia Appendix 5 Risk-of-bias assessment of the included studies.

Multimedia Appendix 6 Differential attrition analysis for the short-term and long-term studies.

Multimedia Appendix 7 Extended results for the exploratory subgroup analysis.

## Abbreviations

AI: artificial intelligence
CA: conversational agent
ECA: embodied conversational agent
ePST: electronic problem-solving treatment
OR: odds ratio
PRISMA: Preferred Reporting Items for Systematic Reviews and Meta-Analyses
RCT: randomized controlled trial

## Data Availability

This systematic review and meta-analysis does not contain primary data. The data sets generated and analyzed during this study are available from the corresponding author upon reasonable request. The data will be made available by emailing the corresponding author.
