# Supplementary material for: Attrition in Conversational Agent–Delivered Mental Health Interventions: Systematic Review and Meta-Analysis
Source: J Med Internet Res. 2024 Feb 27;26:e48168. doi: 10.2196/48168 (PMC10933752; doi:10.2196/48168)
Supplement: Multimedia Appendix 2 [file jmir_v26i1e48168_app2.docx]

# Multimedia Appendix 2: Criteria for study selection and extended meta-analysis method

## Criteria for studies selection

Table 1 describes the inclusion and exclusion criteria of the study.

Table 1. Inclusion and exclusion criteria

| Criteria | Inclusion criteria | Exclusion criteria |
| --- | --- | --- |
| Study Design | - RCT, cRCT, quasi-RCT, cross-over RCT. | - Qualitative study, observation study, cohort study, non-experimental study. |
| Population | - Any age groups. - Healthy population, population with subclinical symptom presentations, clinically diagnosed patients based on DSM or ICD. | - Unable to personally respond to the conversation verbally or textually. |
| Intervention | - CA-delivered intervention with   - synchronous two-way exchanges between CA and user intervention.   - Autonomous response by CA.   - Smartphone-based or computer-based (desktop/laptop).   - Delivered mental health training or psychotherapeutic content to increase mental wellbeing or reduce symptoms of mental health conditions. | - ‘Wizard of Oz’ interventions. - Dialogue system with asynchronous response system. - Studies for the purpose of screening or diagnosis with no intervention components. - Studies with one-time or one session only intervention. |

## Extended meta-analysis method

### Types of outcome measures

The primary outcome is the reported attrition number and the attrition rate calculated by the weighted risk of attrition of participants against the sample size of the studies for participants assigned to the CA intervention who then discontinued the study. This includes total attrition number and attrition in the intervention and comparison groups.

### Data collection and analysis

#### Selection of studies

On updating the searches from 2020 to 2022, we imported all identified references from the different electronic databases into a single file. The duplicated records were removed using revtool on R [1] and manually on Zotero. We performed the screening using ASReview [2], an open source machine learning software. The software utilizes active learning algorithm to actively sort and resort the records by prioritizing the most relevant records first based on user’s inclusion and exclusion decisions. Based on prior simulation studies [2], ASReview saved an average of 83% of screening time to identify about 95% of the relevant studies. One reviewer (AIJ) performed the screening on ASReview after deduplication of the records. We defined the stop screening criteria as having screened at least 33% of the total records and ASReview producing 50 consecutive non-relevant records as recommended by the simulation studies [2].

One reviewer (AIJ) retrieved the full text of the studies, and two reviewers (AIJ and XW) assessed their eligibility independently and in parallel. The disagreements were discussed and resolved between the reviewers and with a third reviewer acting as the arbiter (LM). Studies that were previously identified from our previous reviews (up to 2020) and fit the inclusion criteria of this review were included based on discussions between three reviewers (AIJ, XW, and LM).

#### Data extraction and management

Data were extracted using a data extraction form on Microsoft Excel. The data extraction form was piloted by two reviewers (AIJ and XW) on the same five papers and amended in line with the feedback. We also included additional fields as required from the data extraction form that we referenced from Linardon and Fuller-Tyszkiewicz [3]. Four reviewers (AIJ, XW, GS, and NF) extracted the data independently and in parallel.

We extracted the year of publication, study design, type of comparison group (active or inactive comparison group), type of intervention, details on the CAs including the type of CA (rule-based or AI-enhanced), the personality of the CA [4], level of human support, presence of reminder mechanism and the input and output modalities of the CA. Additionally, we extracted information on the study duration, compensation of study participants and any other mechanism included specifically to increase user engagement. Disagreements between reviewer were resolved by discussion, or by engaging a third review author that will act as an arbiter if the disagreements persist.

#### Assessment of risk of bias in included studies

Four reviewers (AIJ, XW, GS, and NF) independently assessed the risk of bias in the included studies using the Cochrane’s Risk of Bias Tool version 2.0 [5]. The risk of bias assessment was piloted with 10 studies for consistency and clarity of judgement by two reviewers (AIJ and XW). The risk of bias assessed the following domains: random sequence generation; deviation from the intended interventions, missing outcome data, measurement of outcome, and selection of the reported results. We judged the risk of bias for each study as ‘yes’, ‘no’ or ‘unclear’, indicating high, low, or unclear risk of bias respectively following the prompts. Multimedia Appendix 5 reported in the risk of bias table, graph, and summary. Any potential disagreements were resolved by discussion to reach a consensus. We requested clarification or more data from one study author and did not receive any response after sending a reminder 2 weeks later. Assessment of publication bias was reported via funnel plot and Egger test for publication bias [6].

#### Data analysis

The meta-analysis was conducted based on the approach outlined by Linardon and Fuller-Tyszkiewicz [3] and by the Cochrane Handbook [5]. We defined attrition as the number of participants who dropped out of the study during the intervention period by not completing the post-intervention assessment. We did not include the follow-up period [3]. For crossover design studies, we only included the data before the crossover following the above definition. The second part of the crossover was not considered as the follow up period.

The study's overall attrition was estimated by calculating the weighted pooled event rate using random effect models based on meta-proportional approach [5] using Freeman-Tukey double arcsine transformed proportion [7]. This indicated the relative risk of attrition against the sample size of the studies for participants assigned to the CA intervention group. Event rates were then converted to percentages of event per 100 participants and calculated separately for all the included studies, short-term studies (≤8 weeks from baseline) and longer-term studies (>8 weeks from baseline). We used short- and long-term groupings to facilitate comparison between our result and previous study on attrition in smartphone delivered interventions for mental health problems [3].

The differential attrition was calculated as the odds ratio of the likelihood to attrit between the CA-delivered intervention compared to the comparison condition. The odds ratios were calculated using random effect models separately for short-term and long-term studies weighted by their inverse variance. Studies with zero events on both arms were weighted as zero and a correction of 0.5 was added to the arm with zero event as a continuity correction. Log Odds Ratio (log OR) of more than one indicates higher likelihood of attrition in the CA-delivered interventions compared to controls. We also conducted sub-group analyses to explore sources of heterogeneity.

We used the *InfluenceAnalysis and metainf* functions from *dmetar* ver 0.0.9000 to detect outliers in the meta-analysis models [7]. We used the combination of influence diagnostics such as externally standardized residuals, leave-one-out’s τ^2^ and *Q* values, and covariance ration to identify outlying studies [38]. We also plotted the leave-one-out forest plots to identify studies that may distort the attrition rate estimates and precision.

Heterogeneity of effect sizes for both overall and differential attrition was characterized using *I*^2^ and interpreted based on the Cochrane Handbook guidelines [5]. Subgroup analyses were conducted using mixed effect models to explore sources of heterogeneity. We compared: (1) studies with high risk of bias defined as studies with at least one domain identified as “High Risk” using Risk of Bias Tool 2.0 against studies without any domain identified as “High Risk”; (2) studies that reported industry funding against studies that reported public funding sources only; (3) the sub-duration of the studies by comparing studies that lasted 0–4 weeks against those that lasted 5–8 weeks; and studies that lasted 9–12 weeks against studies that lasted more than 13 weeks; (4) studies with RCT design compared to pilot RCT design; (5) the intervention’s target disorders; (6) studies that reported the use of CBT techniques against studies that did not; (7) studies that reported the use of mindfulness techniques against studies that did not; (8) studies that included minimal personalization defined as addressing participants by their given name but with no difference in the content of the intervention, substantial personalization, defined as studies that delivered substantially different content to participants depending on their preference or selection based on the dialogue branching algorithm, major personalization defined as major differences in the language, tone, and delivery of the content between two participants as a result of their preference and selection based on natural language processing and generation; (9) studies that used rule-based algorithm against AI-enhanced CA that included elements of machine learning or natural language processing; (10) the type of CA visualization used by the studies such as avatar only, embodied CA, or no visualization; (11) studies specifying financial incentives against those that did not; (12) studies that included reminders to reengage with the intervention against those that did not; (13) the delivery channel of the intervention comparing web-based applications, computer-based or embedded program that was installed on desktop, laptop, or tablet, smartphone app, and intervention that was delivered on messenger apps such as Slack, Facebook Messenger, or Telegram; (14) studies that included CA-intervention to supplement existing therapy or vice-versa; (15) studies that included in-person enrolment option compared to those with remote options only such as via telephone or online enrolment only; (16) the types of study population specifically at-risk, defined as participants who were screened for mental health conditions but not did not underwent clinical interviews, clinical population, defined as participants who were clinically interviewed for mental health conditions, and general population, defined as participants who were not screened for mental health conditions; (17) studies with pre-defined session length against studies with user-defined session length; (18) studies that included symptoms tracker against those that did not. The next section describes the definition used for data extraction. We used *Q*­_between_ statistics and its associated *p* values [7,8]. We used the *meta* package version 6.1-0 [9] on R to conduct both the overall and differential attrition meta-analyses.

## Data extraction table and definition

| Field | Options | Notes |
| --- | --- | --- |
| Study ID | First author - Year of publication |  |
| Title of article | As reported in paper |  |
| Country | As reported in paper |  |
| Study design | RCT/ Quasi-RCT/ cRCT |  |
| Study aims | As reported in paper |  |
| Target disorder | e.g. Mental wellbeing/depression/anxiety/eating disorder/ADHD | mental well-being indicates that there is no DSM specific target disorder specified in the paper |
| Study Population | At risk/Clinical/General population | At risk indicates that participants were screened in for mental health problems based on self-report (i.e., they were symptomatic), but a mental health condition was not confirmed via a diagnostic interview.   Clinical indicates that all participants were diagnosed with a mental health condition via a semi-structured interview.   General population refers to an unselected sample, whereby no screening for mental health problems was conducted. |
| Intervention information |  |  |
| Type of intervention | Treatment and monitoring/ Education and training | Treatment and monitoring indicates that the study focused on reducing or monitoring specific symptoms   Education and training indicates that the study focused on providing information and/or providing specific training program not specifically focusing on reducing specific symptoms |
| Describe the intervention | As reported in paper |  |
| Contains mindfulness | Yes/No | Yes indicates that the authors of the trial clearly specified that the smartphone intervention included any mindfulness technique (e.g., meditation, body scans etc.) This needed to be explicitly stated in the description of the intervention and cannot be implied.  No indicates that no explicit statement of mindfulness techniques was provided. |
| Contains CBT | Yes/No | Yes indicates that the smartphone intervention was based on the first or second wave cognitive behavioural therapies (see Hayes, Vilatte, Levin & Hildebrandt, 2011 for a description). Note that this does not include the principles of the third-wave behaviour therapies (e.g., acceptance and mindfulness approaches). No indicates that the intervention was not based on the first or second wave behaviour therapies. |
| ACT-based intervention | Yes/No | Yes indicates that the entire smartphone intervention was based on the acceptance and commitment theory and therapy approach (see Hayes, Luoma, Bond, Msuda & Lillis, 2006 for a description).  No indicates that the intervention was not based on ACT. Note that stand-alone mindfulness interventions were not coded as “yes”, as they differ to traditional ACT interventions. |
| Motivational Interviewing | Yes/No | Yes if paper explicitly includes motivational interviewing element for behavioural change |
| Cognitive/attentional training intervention | Yes/No | Yes indicates that the intervention was designed to train any cognitive or attentional process.   No if the intervention was not designed to train cognitive or attentional processes. Note that a “gaming” intervention does not automatically constitute a cognitive training intervention. |
| Gamification | Yes/No | Yes indicates that the intervention contains elements of gamifications |
| Journaling/Self-reflection | Yes/No | Yes indicates that the intervention contains elements of journaling, autobiographical, self-reflection related or not related to CBT |
| Personalization | Yes/No | Yes indicates elements of tailoring any elements of the intervention to the user's profile/preferences/choice. Includes elements of self-directed learning/ability to choose which modules to complete first  No indicates that every user will go through the exact same intervention |
| Degree of personalization | Minimal/Substantial | Minimal indicates just addressing user by name or using user's data but no changes to the actual content of the intervention  Substantial indicates differences between two users in the language, tone, and delivery of the content based on users' preference/selection (rule-based branching personalization)  Major indicates major differences between two users in the language, tone, and delivery of the content based on users' preference/selection (NLG/NLP) |
| Contains wearables | Yes/No | Yes indicates that study includes additional wearable devices beyond primary delivery model |
| Blended design | Yes/No | Yes indicates whether other professional/non-professional involved in the study |
| Type of blended design | professional/non-professional | professional indicates that the blended support was provided by individual with specific certification or qualification such as nurses, doctors, clinical psychologist, counsellors  non-professional indicates general population with or without training |
| Enrolment method | Virtual/telephone/in-person | Virtual indicates that the participant could sign up to the study and complete the entire trial online and without any direct contact with the research team (i.e., a simple mouse click would allow entry to the study). |
| Reminders provided | Yes/No | Yes indicates that the researchers reminded participants to engage, use, or access the smartphone intervention via text messages, emails, or phone calls during the course of the intervention phase.   No indicates that no reminders were offered to participants to engage in the intervention. Note that reminder emails/texts at post-test asking participants to complete the study questionnaires were not coded as “yes”. Instead, the researchers needed to provide reminders with the intention of promoting intervention usage. |
| Mood/Behaviour tracker | Yes/No | Yes indicates that the researcher tracked user's mood or specific behaviour periodically. This does not include trial specific assessment e.g. pre-post test. |
| Offered monetary compensation | Yes/No |  |
| Number of sessions required to complete the intervention | 1/2/3/4/…/ Determined by user (e.g. educational CA may be accessed for different enquiries according to user's need)/ Not specified | If determined by user please indicate the intended use if it is available e.g. complete at least 50% of the modules/programs |
| Duration of CA-User relationship | short-term/long-term/not defined | short-term if the CA immediately responded to user queries in one or a few interactions   long-term if the CA repeatedly interacted with the user over several interdependent sessions. |
| Duration of the study | As reported in paper |  |
| Duration of the study | 1 - 8 weeks / > 8 weeks |  |
| Type of control/comparison | as reported in paper | e.g. wait-list, psychoeducation, cbt without dhi |
| Type of control/comparison | wait-list/treatment as usual/active control |  |
| Conversational agents (CAs) information |  |  |
| CA name | As reported |  |
| Availability | App store/ Research participants only |  |
| Self-consent | Yes/No | Yes indicates that participants self-consented to be part of the study  No indicates that a 3rd-party consented on behalf of participants to be part of the study |
| Delivery channel (Accessibility of chatbot) | Smartphone apps/ Web based/ Desktop computer based/ Smartphone-embedded software (Siri,Google Assistant,Alexa,etc)/ Telegram/ WeChat/ SMS-MMS/ Windows live messenger/ Facebook Messenger/ Not specified |  |
| Type of chatbot (level of intelligence) | Rule based/ AI-enhanced (NLP/NLG included) | Rule-based indicates that all the responses and choices are predetermined  AI-enhanced indicates that some elements of NLP/NLG or AI algorithm included in the intervention |
| Type of chatbot | ECA/ Not ECA but avatar/ Not ECA nor avatar |  |
| Modality of dialoge creation | Predetermined text/ Free text (NLP/AI based combo)/ Both/ Not specified |  |
| Input modalities  (input all that apply) | Text/ Voice/ Images/ Video/ Not specified |  |
| Output modalities (input all that apply) | Text/ Voice/ Images/ Video/ Not specified |  |
| Chatbot personality (input all that apply) | Coach like/ Health care professional like  Conversational agent identity/ Human like Gender specific/ Culture specific  Informal/ Knowledgeable Factual | **Coach like:** encouraging, motivating, and nurturing **Health care professional like**: mimics a health care professional **Conversational agent identity: e**xplicitly identifies as a CA **Human like**: emulates humans **Gender specific**: Male and female versions available **Culture specific:**  speaks the native language or has native names **Informal**: like talking to a friend. Uses exclamations, abbreviations, and emoticons **Knowledgeable**: content created or informed by medical experts **Factual:** nonjudgmental, no personal opinions, and responses based on facts or observations |
| Goal of the chatbot | As reported in paper |  |
| Method of data collection |  |  |
| Primary outcomes | As reported in paper |  |
| Secondary outcomes | As reported in paper |  |
| Pre-intervention assessment | Yes/No | with or without baseline assessment |
| Post-intervention assessment | Yes/No | with or without post-test assessment |
| Follow-up assessment | Yes/No | with or without follow-up assessment |
| Follow-up duration | As reported | Duration between post-test and follow-up |
| EMA/periodic assessment | Yes/No | with or without EMA/periodic |
| Duration of EMA | As reported | Duration between assessment e.g. daily, weekly, monthly |
| No of times outcome assessments | Pre-post: 2, Pre-post-followup: 3 EMA: daily, weekly | include all points of outcome measurement assessment if there are multiple assessment used |
| Total number of outcomes measures tools | As reported |  |
| How were the outcomes measured? | Self-reported/ Objectively measured |  |
| What platform were used to collect the outcomes | online survey platform/chatbot/pen-and-paper/in-app data collection | Online survey platform includes google form, qualtrics, survey monkey, limesurvey  chatbot refers to data collection via the app with the chatbot asking users the survey questions specifically  in-app data collection refers to surveys/questionnaire collected in app and not via 3rd party survey platforms |
| Attrition Data |  |  |
| Protocol link | Yes/No |  |
| Registered | Yes/No |  |
| Missing Data Handling procedure | Row-wise deletion/mean/median/mode/multiple/model-based imputation |  |
| Per Protocol analysis | Yes/No | removed participants who did not adhere |
| Intention to Treat analysis | Yes/No | same number of participants as assigned |
| Age range |  |  |
| Age mean & SD |  |  |
| Gender |  | number of males, number of females |
| Education |  |  |
| Income level |  |  |
| Total number of participants | As reported | # participants in at the start after randomization |
| # intervention participants | As reported | # participants in intervention group at the start (if there are more than one intervention group, combine the total number of the groups) |
| # control participants | As reported | # participants in the comparison group at the start |
| total of attrition | As reported | # participants who dropped out |
| # intervention at end of study | As reported | # participants at the end of study |
| # control at end of study | As reported | # participants at the end of study |
| # intervention attrition | As reported | # attrited participants at the end of study |
| # control attrition | As reported | # attrited participants at the end of study |
| Reason for dropout | As reported | list all the reason reported |
| Engagement data | as reported | average number of logins, number of conversation turns, hours spent on app, days active on app |
| Effectiveness of the intervention | As reported in paper |  |
| Type of funding source | industry sponsorship/ public and industry sponsorship/public funding only |  |
| Funding name | as reported |  |

# References

1. Westgate MJ. revtools: An R package to support article screening for evidence synthesis. Research Synthesis Methods 2019;10(4):606–614. doi: 10.1002/jrsm.1374

2. van de Schoot R, de Bruin J, Schram R, Zahedi P, de Boer J, Weijdema F, Kramer B, Huijts M, Hoogerwerf M, Ferdinands G, Harkema A, Willemsen J, Ma Y, Fang Q, Hindriks S, Tummers L, Oberski DL. An open source machine learning framework for efficient and transparent systematic reviews. Nat Mach Intell Nature Publishing Group; 2021 Feb;3(2):125–133. doi: 10.1038/s42256-020-00287-7

3. Linardon J, Fuller-Tyszkiewicz M. Attrition and adherence in smartphone-delivered interventions for mental health problems: A systematic and meta-analytic review. J Consult Clin Psychol 2020 Jan;88(1):1–13. PMID:31697093

4. Tudor Car L, Dhinagaran DA, Kyaw BM, Kowatsch T, Joty S, Theng Y-L, Atun R. Conversational Agents in Health Care: Scoping Review and Conceptual Analysis. J Med Internet Res 2020 Aug 7;22(8):e17158. PMID:32763886

5. Higgins J, Thomas J, Chandler J, Cumpston M, Li T, Page MJ. Cochrane Handbook for Systematic Reviews of Interventions version 6.3 (updated February 2022). Welch V, editor. Cochrane; 2022. Available from: www.training.cochrane.org/handbook

6. Egger M, Smith GD, Schneider M, Minder C. Bias in meta-analysis detected by a simple, graphical test. BMJ British Medical Journal Publishing Group; 1997 Sep 13;315(7109):629–634. PMID:9310563

7. Harrer M, Cuijpers P, Furukawa TA, Ebert DD. Doing Meta-Analysis in R. Available from: https://bookdown.org/MathiasHarrer/Doing_Meta_Analysis_in_R/ [accessed Feb 27, 2023]

8. Borenstein M, editor. Introduction to meta-analysis. Chichester, U.K: John Wiley & Sons; 2009. ISBN:978-0-470-05724-7

9. Schwarzer G. meta: General Package for Meta-Analysis. 2023. Available from: https://CRAN.R-project.org/package=meta [accessed Mar 2, 2023]
