# Supplementary material for: Attrition in Conversational Agent–Delivered Mental Health Interventions: Systematic Review and Meta-Analysis
Source: J Med Internet Res. 2024 Feb 27;26:e48168. doi: 10.2196/48168 (PMC10933752; doi:10.2196/48168)
Supplement: Multimedia Appendix 4 [file jmir_v26i1e48168_app4.docx]

# Multimedia Appendix 4: Tables of excluded studies.

Table 1: Excluded studies from database searches up to June 2022

| Reference | Reason for exclusion |
| --- | --- |
| [1] | Missing attrition data |
| [2,3] | Conference abstract only |
| [4–11] | Feature testing |
| [12–68] | Studies not focusing on mental health outcomes |
| [69–102] | Not RCT |
| [103–109] | Duplicates |

Table 2: Excluded studies from citation searches

| References | Reason for exclusion |
| --- | --- |
| [110,111] | Published after search date |
| [112,113] | Not English |
| [114–134] | Not conversational agent |
| [135] | Clinical outcome |
| [136,137] | Not mental health |
| [138] | Reanalysis of an included study |
| [102,139–150] | Duplicates |

# References

1. Dulin P, Mertz R, Edwards A, King D. Contrasting a Mobile App With a Conversational Chatbot for Reducing Alcohol Consumption: Randomized Controlled Pilot Trial. JMIR Form Res 2022 May 16;6(5):e33037. PMID:35576569

2. Lee C.H. Application of Chatbot for patients with methamphetamine use disorder: A preliminary randomized controlled trial. Asia-Pacific Psychiatry Netherlands: Wiley-Blackwell; 2021;13(SUPPL 1). doi: 10.1111/appy.12461

3. Oh J. Chatbot and VR-based cognitive behavioral treatment for panic disorder. Asia-Pacific Psychiatry Netherlands: Wiley-Blackwell; 2021;13(SUPPL 1). doi: 10.1111/appy.12459

4. Abdulrahman A, Richards D, Bilgin AA. Changing users’ health behaviour intentions through an embodied conversational agent delivering explanations based on users’ beliefs and goals. Behaviour & Information Technology 2022; doi: 10.1080/0144929X.2022.2073269

5. Gonzalez DAZ, Richards D, Bilgin AA. Making it Real: A Study of Augmented Virtuality on Presence and Enhanced Benefits of Study Stress Reduction Sessions. INTERNATIONAL JOURNAL OF HUMAN-COMPUTER STUDIES 2021 Mar;147. doi: 10.1016/j.ijhcs.2020.102579

6. He L, Basar E, Wiers RW, Antheunis ML, Krahmer E. Can chatbots help to motivate smoking cessation? A study on the effectiveness of motivational interviewing on engagement and therapeutic alliance. BMC Public Health 2022 Apr 12;22(1):726. PMID:35413887

7. Loveys K, Sagar M, Zhang X, Fricchione G, Broadbent E. Effects of Emotional Expressiveness of a Female Digital Human on Loneliness, Stress, Perceived Support, and Closeness Across Genders: Randomized Controlled Trial. J Med Internet Res 2021 Nov 25;23(11):e30624. PMID:34842540

8. Matsumoto A, Kamita T, Tawaratsumida Y, Nakamura A, Fukuchimoto H, Mitamura Y, Suzuki H, Munakata T, Inoue T. Combined Use of Virtual Reality and a Chatbot Reduces Emotional Stress More Than Using Them Separately. JOURNAL OF UNIVERSAL COMPUTER SCIENCE 2021;27(12):1371–1389. doi: 10.3897/jucs.77237

9. Meng J, Dai Y. Emotional Support from AI Chatbots: Should a Supportive Partner Self-Disclose or Not? Journal of Computer-Mediated Communication 2021;26(4):207–222. doi: 10.1093/jcmc/zmab005

10. Nelekar S, Abdulrahman A, Gupta M, Richards D. Effectiveness of embodied conversational agents for managing academic stress at an Indian University (ARU) during COVID-19. BRITISH JOURNAL OF EDUCATIONAL TECHNOLOGY 2022 May;53(3):491–511. doi: 10.1111/bjet.13174

11. Shidara KT Hiroki; Adachi, Hiroyoshi; Kanayama, Daisuke; Sakagami, Yukako; Kudo, Takashi; Nakamura, Satoshi. Automatic Thoughts and Facial Expressions in Cognitive Restructuring With Virtual Agents. Frontiers in Computer Science 2022;4(NA):NA-NA. doi: 10.3389/fcomp.2022.762424

12. Alghamdi E, Alnanih R. Chatbot Design for a Healthy Life to Celiac Patients: A Study According to a New Behavior Change Model. INTERNATIONAL JOURNAL OF ADVANCED COMPUTER SCIENCE AND APPLICATIONS 2021 Oct;12(10):698–707.

13. Altay S, Schwartz M, Hacquin AS, Allard A, Blancke S, Mercier H. Scaling up interactive argumentation by providing counterarguments with a chatbot. Nat Hum Behav 20220214th ed 2022 Apr;6(4):579–592. doi: 10.1038/s41562-021-01271-w

14. Anan T, Kajiki S, Oka H, Fujii T, Kawamata K, Mori K, Matsudaira K. Effects of an Artificial Intelligence-Assisted Health Program on Workers With Neck/Shoulder Pain/Stiffness and Low Back Pain: Randomized Controlled Trial. JMIR Mhealth Uhealth 20210924th ed 2021 Sep 24;9(9):e27535. doi: 10.2196/27535

15. Apergi LA, Bjarnadottir MV, Baras JS, Golden BL, Anderson KM, Chou JL, Shara N. Voice Interface Technology Adoption by Patients With Heart Failure: Pilot Comparison Study. JMIR MHEALTH AND UHEALTH 2021 Apr 1;9(4). doi: 10.2196/24646

16. Arguedas M, Daradoumis T. Analysing the role of a pedagogical agent in psychological and cognitive preparatory activities. Journal of computer assisted learning 2021;37(4):1167‐1180. doi: 10.1111/jcal.12556

17. Asensio-Cuesta S, Blanes-Selva V, Conejero A, Portolés M, García-Gómez M. A user-centered chatbot to identify and interconnect individual, social and environmental risk factors related to overweight and obesity. Inform Health Soc Care 20210525th ed 2022 Jan 2;47(1):38–52. doi: 10.1080/17538157.2021.1923501

18. Asensio-Cuesta S, Blanes-Selva V, Portoles M, Conejero JA, Garcia-Gomez JM. How the Wakamola chatbot studied a university community’s lifestyle during the COVID-19 confinement. HEALTH INFORMATICS JOURNAL 2021 Apr;27(2). doi: 10.1177/14604582211017944

19. Bassi G, Giuliano C, Perinelli A, Forti S, Gabrielli S, Salcuni S. A Virtual Coach (Motibot) for Supporting Healthy Coping Strategies Among Adults With Diabetes: Proof-of-Concept Study. JMIR Hum Factors 20220121st ed 2022 Jan 21;9(1):e32211. doi: 10.2196/32211

20. Beinema T, Op den Akker H, Hurmuz M, Jansen-Kosterink S, Hermens H. Automatic topic selection for long-term interaction with embodied conversational agents in health coaching: A micro-randomized trial. Internet Interv 2022 Mar;27:100502. PMID:35198412

21. Bickmore T, Zhang Z, Reichert M, Julce C, Jack B. Promotion of Preconception Care Among Adolescents and Young Adults by Conversational Agent. Journal of adolescent health 2020;67(2S):S45‐S51. doi: 10.1016/j.jadohealth.2019.09.006

22. Block VJ, Gopal A, Rowles W, Yueh C, Gelfand JM, Bove R. CoachMS, an innovative closed-loop, interdisciplinary platform to monitor and proactively treat MS symptoms: a pilot study. Multiple sclerosis journal - experimental, translational and clinical 2021;7(1). doi: 10.1177/2055217321988937

23. Calvaresi D, Calbimonte JP, Siboni E, Eggenschwiler S, Manzo G, Hilfiker R, Schumacher M. EREBOTS: Privacy-Compliant Agent-Based Platform for Multi-Scenario Personalized Health-Assistant Chatbots. ELECTRONICS 2021 Mar;10(6). doi: 10.3390/electronics10060666

24. Colwell N, Sheppard A, Egan J. Digital therapautic effective in managing metabolic syndrome parameters. European Heart Journal 2021;42(SUPPL 1):3104. doi: 10.1093/eurheartj/ehab724.3104

25. Corbett CF, Combs EM, Chandarana PS, Stringfellow I, Worthy K, Nguyen T, Wright PJ, O’Kane JM. Medication Adherence Reminder System for Virtual Home Assistants: Mixed Methods Evaluation Study. JMIR Form Res 20210713th ed 2021 Jul 13;5(7):e27327. doi: 10.2196/27327

26. De Marchi F, Amantea IA, Serioli M, Sulis E, Boella G, Alloatti F, Bolioli A, Riso S, Cantello R, Mazzini L. E-health solutions for amyotrophic lateral sclerosis patients: A chatbot for dietary monitoring. Journal of the Neurological Sciences 2021;429(Supplement):119386. doi: 10.1016/j.jns.2021.119386

27. Dhinagaran DA, Sathish T, Soong A, Theng YL, Best J, Tudor Car L. Conversational Agent for Healthy Lifestyle Behavior Change: Web-Based Feasibility Study. JMIR Form Res 20211203rd ed 2021 Dec 3;5(12):e27956. doi: 10.2196/27956

28. Du C. Assistant Training System of Teenagers’ Physical Ability Based on Artificial Intelligence. MATHEMATICAL PROBLEMS IN ENGINEERING 2021 Mar 13;2021. doi: 10.1155/2021/5526509

29. Dupuy L, Morin CM, de Sevin E, Taillard J, Salles N, Bioulac S, Auriacombe M, Micoulaud‐Franchi J, Philip P. Smartphone‐based virtual agents and insomnia management: A proof‐of‐concept study for new methods of autonomous screening and management of insomnia symptoms in the general population. Journal of Sleep Research 2022;31(2). doi: 10.1111/jsr.13489

30. Echeazarra L, Pereira J, Saracho R. TensioBot: a Chatbot Assistant for Self-Managed in-House Blood Pressure Checking. Journal of medical systems 2021;45(4):54. doi: 10.1007/s10916-021-01730-x

31. Fazlollahi A, Bakhaidar M, Alsayegh A, Yilmaz R, Winkler-Schwartz A, Langleben I, Mirchi N, Ledwos N, Harley J, Del Maestro R. Artificial Intelligence Tutoring Compared with Expert Instruction in Neurosurgical Simulation Training: a Randomized Controlled Trial. Clinical neurosurgery 2022;68(SUPPL 1):128‐129.

32. Fazlollahi AM, Bakhaidar M, Alsayegh A, Yilmaz R, Winkler-Schwartz A, Mirchi N, Langleben I, Ledwos N, Sabbagh AJ, Bajunaid K, et al. Effect of Artificial Intelligence Tutoring vs Expert Instruction on Learning Simulated Surgical Skills Among Medical Students: a Randomized Clinical Trial. JAMA network open 2022;5(2):e2149008. doi: 10.1001/jamanetworkopen.2021.49008

33. Gardiner P, Bickmore T, Yinusa-Nyahkoon L, Reichert M, Julce C, Sidduri N, Martin-Howard J, Woodhams E, Aryan J, Zhang Z, et al. Using Health Information Technology to Engage African American Women on Nutrition and Supplement Use During the Preconception Period. Frontiers in endocrinology 2020;11:571705. doi: 10.3389/fendo.2020.571705

34. Geoghegan L, Harrison C, Collins D, Gardiner M, Rodrigues J. Optimising outpatient care after paediatric burns: The development of a chatbot to identify complications and provide clinical advice. British Journal of Surgery 2021;108(SUPPL 6). doi: 10.1093/bjs/znab258.046

35. Hendawi R, Li J, Alian S. ADDietCoach: A Personalized Virtual Diet Coach for Alzheimer’s Disease. INTERNATIONAL JOURNAL OF E-HEALTH AND MEDICAL COMMUNICATIONS 2021 Nov;12(6):1–18. doi: 10.4018/IJEHMC.20211101.oa6

36. Hong YJ, Piao M, Kim J, Lee JH. Development and Evaluation of a Child Vaccination Chatbot Real-Time Consultation Messenger Service during the COVID-19 Pandemic. APPLIED SCIENCES-BASEL 2021 Dec;11(24). doi: 10.3390/app112412142

37. Hurmuz MZM, Jansen-Kosterink SM, Beinema T, Fischer K, Op den Akker H, Hermens HJ. Evaluation of a virtual coaching system eHealth intervention: A mixed methods observational cohort study in the Netherlands. Internet Interv 20220205th ed 2022 Mar;27:100501. doi: 10.1016/j.invent.2022.100501

38. Jones R, Chen K, Kostenko E, DeMaria A, Su B, Villa A, Swarup M, Weida J, Tuuli M. Patient education for prenatal aneuploidy testing using a chatbot: a multicenter randomized controlled trial. Molecular Genetics and Metabolism 2021;132(Supplement 1):S309. doi: 10.1016/S1096-7192%2821%2900558-8

39. Kataoka Y, Takemura T, Sasajima M, Katoh N. Development and Early Feasibility of Chatbots for Educating Patients With Lung Cancer and Their Caregivers in Japan: Mixed Methods Study. JMIR CANCER 2021 Mar 10;7(1). doi: 10.2196/26911

40. Kobayashi T, Nishina Y, Tomoi H, Harada K, Matsumoto E, Inaba K, Ishihara J, Sasaki S, Horimukai K, Seguchi K, Tanaka K, Takahashi H, Salinas JL, Yamada Y. Corowa-kun: Impact of a COVID-19 Vaccine Information Chatbot on Vaccine Hesitancy, Japan 2021. Open Forum Infectious Diseases 2021;8(SUPPL 1):S321–S322. doi: 10.1093/ofid/ofab466.638

41. Kowatsch T, Schachner T, Harperink S, Barata F, Dittler U, Xiao G, Stanger C, Wangenheim FV, Fleisch E, Oswald H, Moller A. Conversational Agents as Mediating Social Actors in Chronic Disease Management Involving Health Care Professionals, Patients, and Family Members: Multisite Single-Arm Feasibility Study. JOURNAL OF MEDICAL INTERNET RESEARCH 2021 Feb 17;23(2). doi: 10.2196/25060

42. Kramer LL, van Velsen L, Clark JL, Mulder BC, de Vet E. Use and Effect of Embodied Conversational Agents for Improving Eating Behavior and Decreasing Loneliness Among Community-Dwelling Older Adults: Randomized Controlled Trial. JMIR Form Res 20220411th ed 2022 Apr 11;6(4):e33974. doi: 10.2196/33974

43. Krieger JL, Neil JM, Duke KA, Zalake MS, Tavassoli F, Vilaro MJ, Wilson-Howard DS, Chavez SY, Laber EB, Davidian M, et al. A Pilot Study Examining the Efficacy of Delivering Colorectal Cancer Screening Messages via Virtual Health Assistants. American journal of preventive medicine 2021;61(2):251‐255. doi: 10.1016/j.amepre.2021.01.014

44. Krishnakumar A, Verma R, Chawla R, Sosale A, Saboo B, Joshi S, M. Shaikh, Shah A, Kolwankar S, Mattoo V. Evaluating Glycemic Control in Patients of South Asian Origin With Type 2 Diabetes Using a Digital Therapeutic Platform: Analysis of Real-World Data. JOURNAL OF MEDICAL INTERNET RESEARCH 2021 Mar 25;23(3). doi: 10.2196/17908

45. Krivanek T, McFeeley B, Nicastri CM, Babazadeh D, Daffner KR, Gale SA. The Brain Health Champion study: a health coaching intervention with mobile technology in older adults with mild cognitive impairment or risk factors for dementia. Alzheimer’s & dementia 2021;17:e054068‐. doi: 10.1002/alz.054068

46. Ma DC, Potters L, Lee L, Bloom BF, Andrews JZ, Chen W, Teckie S. Patient-Reported Outcomes Using Automated Chatbot for Breast Cancer Patients Receiving Radiation Therapy. International Journal of Radiation Oncology Biology Physics 2021;111(3 Supplement):e96. doi: 10.1016/j.ijrobp.2021.07.484

47. Ma D, Orner D, Ghaly MM, Parashar B, Ames JW, Chen WC, Potters L, Teckie S. Automated health chats for symptom management of head and neck cancer patients undergoing radiation therapy. Oral Oncol 20211023rd ed 2021 Nov;122:105551. doi: 10.1016/j.oraloncology.2021.105551

48. Maeda E, Miyata A, Boivin J, Nomura K, Kumazawa Y, Shirasawa H, Saito H, Terada Y. Promoting fertility awareness and preconception health using a chatbot: A randomized controlled trial. Human Reproduction 2020;35(SUPPL 1). Available from: https://ovidsp.ovid.comremotexs.ntu.edu.sg/ovidweb.cgi?T=JS&PAGE=reference&D=emexb&NEWS=N&AN=637628961

49. Mitchell EG. Enabling automated, conversational health coaching with human-centered artificial intelligence. ProQuest Information & Learning; 2022. Available from: https://search.ebscohost.com/login.aspx?direct=true&db=psyh&AN=2022-13252-033&site=ehost-live

50. Murali P, Trinh H, Ring L, Bickmore T, Assoc Comp M. A Friendly Face in the Crowd: Reducing Public Speaking Anxiety with an Emotional Support Agent in the Audience. 2021. p. 156–163. doi: 10.1145/3472306.3478364

51. Nam KH, Kim DY, Kim DH, Lee JH, Lee JI, Kim MJ, Park JY, Hwang JH, Yun SS, Choi BK, Kim MG, Han IH. Conversational Artificial Intelligence for Spinal Pain Questionnaire: Validation and User Satisfaction. Neurospine 20220512th ed 2022 May 12; doi: 10.14245/ns.2143080.540

52. Napolitano MA, Harrington CB, Patchen L, Ellis LP, Ma T, Chang K, Gaminian A, Bailey CP, Evans WD. Feasibility of a digital intervention to promote healthy weight management among postpartum african american/black women. International journal of environmental research and public health 2021;18(4):1‐16. doi: 10.3390/ijerph18042178

53. Nct. Using Artificial Intelligence-based ChatBot to Improve Women’s Participation to Cervical Cancer Screening Programme. https://clinicaltrials.gov/show/NCT05286034 2022; Available from: https://www.cochranelibrary.com/central/doi/10.1002/central/CN-02391419/full

54. Ogawa M, Oyama G, Morito K, Kobayashi M, Yamada Y, Shinkawa K, Kamo H, Hatano T, Hattori N. Can AI make people happy? The effect of AI-based chatbot on smile and speech in Parkinson’s disease. Parkinsonism Relat Disord 20220505th ed 2022 May 5;99:43–46. doi: 10.1016/j.parkreldis.2022.04.018

55. Pirolli P, Youngblood GM, Du HL, Konrad A, Nelson L, Springer A. Scaffolding the Mastery of Healthy Behaviors with Fittle plus Systems: Evidence-Based Interventions and Theory. HUMAN-COMPUTER INTERACTION 2021 Mar 4;36(2):73–106. doi: 10.1080/07370024.2018.1512414

56. Rabinowitz A, Collier G. Development of a Conversational Agent for Promoting Increased Activity in Users with Traumatic Brain Injury. Archives of Physical Medicine and Rehabilitation 2021;102(10):e83. doi: 10.1016/j.apmr.2021.07.719

57. Ris K, Janssen J, Beckeringh JJ, Hugtenburg J. The app “Robin” including virtual assistance and a reminder function improves medication adherence in chronic myeloid leukemia patients. International Journal of Clinical Pharmacy 2021;43(3):797. doi: 10.1007/s11096-021-01269-4

58. Röbbelen A, Schmieding ML, Kopka M, Balzer F, Feufel MA. Interactive Versus Static Decision Support Tools for COVID-19: Randomized Controlled Trial. JMIR Public Health Surveill 20220415th ed 2022 Apr 15;8(4):e33733. doi: 10.2196/33733

59. Schreuter D, van der Putten P, Lamers MH. Trust me on this one: Conforming to conversational assistants. Minds and Machines: Journal for Artificial Intelligence, Philosophy and Cognitive Science 2021;31(4):535–562. doi: 10.1007/s11023-021-09581-8

60. Spencer E, Phalore J, Bickmore T, Kim S, Jang H, Gogoi R. Relational agents in cervical cancer education: A pilot study to determine acceptability and impact of interactive education on vaccine advocacy. International Journal of Gynecological Cancer 2021;31(SUPPL 4):A120–A121. doi: 10.1136/ijgc-2021-IGCS.303

61. Stasinaki A, Büchter D, Shih CI, Heldt K, Güsewell S, Brogle B, Farpour-Lambert N, Kowatsch T, l’Allemand D. Effects of a novel mobile health intervention compared to a multi-component behaviour changing program on body mass index, physical capacities and stress parameters in adolescents with obesity: a randomized controlled trial. BMC Pediatr 20210709th ed 2021 Jul 9;21(1):308. doi: 10.1186/s12887-021-02781-2

62. Stoevesandt D, Jahn P, Watzke S, Wohlgemuth WA, Behr D, Buhtz C, Faber I, Enger S, Schwarz K, Brill R. Comparison of Acceptance and Knowledge Transfer in Patient Information Before an MRI Exam Administered by Humanoid Robot Versus a Tablet Computer: A Randomized Controlled Study. Rofo 20210610th ed 2021 Aug;193(8):947–954. doi: 10.1055/a-1382-8482

63. Sy B, Wassil M, Connelly H, Hassan A. Behavioral Predictive Analytics Towards Personalization for Self-management: a Use Case on Linking Health-Related Social Needs. SN Comput Sci 20220423rd ed 2022;3(3):237. doi: 10.1007/s42979-022-01092-2

64. To QG, Green C, Vandelanotte C. Feasibility, Usability, and Effectiveness of a Machine Learning-Based Physical Activity Chatbot: Quasi-Experimental Study. JMIR Mhealth Uhealth 20211126th ed 2021 Nov 26;9(11):e28577. doi: 10.2196/28577

65. Tsai WS, Lun D, Carcioppolo N, Chuan CH. Human versus chatbot: Understanding the role of emotion in health marketing communication for vaccines. Psychol Mark 20210727th ed 2021 Jul 27; doi: 10.1002/mar.21556

66. Verma R, Adhikary R, Kolwankar S, Tahilramani L, Motwani P, Pawar S, Bhatt J, Shah A. CARDIOVASCULAR RISK REDUCTION IN PATIENTS WITH HYPERTENSION USING A DIGITAL THERAPEUTICS PLATFORM. Journal of the American College of Cardiology 2021;77(18 Supplement 1):3215. doi: 10.1016/S0735-1097%2821%2904570-8

67. Wu YS, Wang WY, Chan TC, Chiu YL, Lin HC, Chang YT, Wu HY, Liu TC, Chuang YC, Wu J, Chang WY, Sun CA, Lin MC, Tseng VS, Hu JM, Li YK, Hsiao PJ, Chen CW, Kao HY, Lee CC, Hsieh CB, Wang CH, Chu CM. Effect of the Nintendo Ring Fit Adventure Exergame on Running Completion Time and Psychological Factors Among University Students Engaging in Distance Learning During the COVID-19 Pandemic: Randomized Controlled Trial. JMIR Serious Games 20220322nd ed 2022 Mar 22;10(1):e35040. doi: 10.2196/35040

68. Yokomizo R, Nakamura A, Sato M, Nasu R, Hine M, Urayama KY, Kishi H, Sago H, Okamoto A, Umezawa A. Smartphone application improves fertility treatmentrelated literacy: a large-scale surveillance and randomized controlled trial in Japan. Human reproduction (Oxford, England) 2021;36(SUPPL 1):i103‐. doi: 10.1093/humrep/deab127.088

69. Abreu C, Campos PF. Raising Awareness of Smartphone Overuse among University Students: A Persuasive Systems Approach. INFORMATICS-BASEL 2022 Mar;9(1). doi: 10.3390/informatics9010015

70. Albright G, Khalid N, Shockley K, Robinson K, Hughes K, Pace-Danley B. Innovative Virtual Role Play Simulations for Managing Substance Use Conversations: Pilot Study Results and Relevance During and After COVID-19. JMIR Form Res 20210429th ed 2021 Apr 29;5(4):e27164. doi: 10.2196/27164

71. Auriacombe M, Fournet L, Dupuy L, Micoulaud-Franchi JA, de Sevin E, Moriceau S, Baillet E, Alexandre JM, Serre F, Philip P. Effectiveness and Acceptance of a Smartphone-Based Virtual Agent Screening for Alcohol and Tobacco Problems and Associated Risk Factors During COVID-19 Pandemic in the General Population. Frontiers in Psychiatry 2021;12:693687. doi: 10.3389/fpsyt.2021.693687

72. Aymerich-Franch L, Ferrer I. Investigating the use of speech-based conversational agents for life coaching. International Journal of Human-Computer Studies 2022;159:1–8. doi: 10.1016/j.ijhcs.2021.102745

73. Beatty C, Malik T, Meheli S, Sinha C. Evaluating the Therapeutic Alliance with a Free-Text CBT Conversational Agent (Wysa): A Mixed-Methods Study. Frontiers in Digital Health 2022;4. PMID:35480848

74. Ben Abdessalem H, Ai Y, Swamy KSM, Frasson C. Virtual Reality Zoo Therapy for Alzheimer’s Disease Using Real-Time Gesture Recognition. GENEDIS 2020: COMPUTATIONAL BIOLOGY AND BIOINFORMATICS 2021. p. 97–105. doi: 10.1007/978-3-030-78775-2_12

75. Castro L, Baracas L, Hashioka G, Carvalho A. DCBT-I with chatbot and artificial intelligence: A feasibility study in Brazil. Sleep 2021;44(SUPPL 2):A149–A150. doi: 10.1093/sleep/zsab072.375

76. Chung K, Cho HY, Park JY. A Chatbot for Perinatal Women’s and Partners’ Obstetric and Mental Health Care: Development and Usability Evaluation Study. JMIR MEDICAL INFORMATICS 2021 Mar;9(3). doi: 10.2196/18607

77. Darcy A, Daniels J, Salinger D, Wicks P, Robinson A. Evidence of Human-Level Bonds Established With a Digital Conversational Agent: Cross-sectional, Retrospective Observational Study. JMIR Form Res 20210511th ed 2021 May 11;5(5):e27868. doi: 10.2196/27868

78. Gabrielli S, Rizzi S, Bassi G, Carbone S, Maimone R, Marchesoni M, Forti S. Engagement and Effectiveness of a Healthy-Coping Intervention via Chatbot for University Students During the COVID-19 Pandemic: Mixed Methods Proof-of-Concept Study. JMIR Mhealth Uhealth 20210528th ed 2021 May 28;9(5):e27965. doi: 10.2196/27965

79. Heffner JL, Watson NL, Serfozo E, Kelly MM, Reilly ED, Kim D, Baker K, Scout NFN, Karekla M. An Avatar-Led Digital Smoking Cessation Program for Sexual and Gender Minority Young Adults: Intervention Development and Results of a Single-Arm Pilot Trial. JMIR Form Res 20210730th ed 2021 Jul 30;5(7):e30241. doi: 10.2196/30241

80. Jones VK, Hanus M, Yan C, Shade MY, Blaskewicz Boron J, Maschieri Bicudo R. Reducing Loneliness Among Aging Adults: The Roles of Personal Voice Assistants and Anthropomorphic Interactions. Front Public Health 20211210th ed 2021;9:750736. doi: 10.3389/fpubh.2021.750736

81. Kaywan P, Ahmed K, Miao Y, Ibaida A, Gu B. DEPRA: An Early Depression Detection Analysis Chatbot. 2021. p. 193–204. doi: 10.1007/978-3-030-90885-0_18

82. Ko JL, Yuan C, Malherbe B, Yang CH, Kuo PY. Exploring the Effect of Activity Intervention on Reducing Social Media Use: Lessons Learned in a Field Study. 2021. p. 368–374. doi: 10.1007/978-3-030-90179-0_47

83. Leo AJ, Schuelke MJ, Hunt DM, Metzler JP, Miller JP, Areán PA, Armbrecht MA, Cheng AL. A Digital Mental Health Intervention in an Orthopedic Setting for Patients With Symptoms of Depression and/or Anxiety: Feasibility Prospective Cohort Study. JMIR Form Res 20220221st ed 2022 Feb 21;6(2):e34889. doi: 10.2196/34889

84. Leo AJ, Schuelke MJ, Hunt DM, Miller JP, Areán PA, Cheng AL. Digital Mental Health Intervention Plus Usual Care Compared With Usual Care Only and Usual Care Plus In-Person Psychological Counseling for Orthopedic Patients With Symptoms of Depression or Anxiety: Cohort Study. JMIR Form Res 20220504th ed 2022 May 4;6(5):e36203. doi: 10.2196/36203

85. Mariamo AT Caroline E; Léger, Pierre-Majorique; Sénécal, Sylvain; Lau, Marianne Alexandra. Emotional Reactions and Likelihood of Response to Questions Designed for a Mental Health Chatbot Among Adolescents: Experimental Study. JMIR human factors 2021;8(1):e24343-NA. doi: 10.2196/24343

86. Mauriello ML, Tantivasadakarn N, Mora-Mendoza MA, Lincoln ET, Hon G, Nowruzi P, Simon D, Hansen L, Goenawan NH, Kim J, Gowda N, Jurafsky D, Paredes PE. A Suite of Mobile Conversational Agents for Daily Stress Management (Popbots): Mixed Methods Exploratory Study. JMIR Form Res 20210914th ed 2021 Sep 14;5(9):e25294. doi: 10.2196/25294

87. Meheli S, Sinha C, Kadaba M. Understanding People With Chronic Pain Who Use a Cognitive Behavioral Therapy-Based Artificial Intelligence Mental Health App (Wysa): Mixed Methods Retrospective Observational Study. JMIR Hum Factors 20220427th ed 2022 Apr 27;9(2):e35671. doi: 10.2196/35671

88. Miura CC Sinan; Saiki, Sachio; Nakamura, Masahide; Yasuda, Kiyoshi. Assisting Personalized Healthcare of Elderly People: Developing a Rule-Based Virtual Caregiver System Using Mobile Chatbot. Sensors (Basel, Switzerland) 2022;22(10):3829–3829. doi: 10.3390/s22103829

89. Morales-de-Jesús V, Gómez-Adorno H, Somodevilla-García M, Vilariño D. Conversational System as Assistant Tool in Reminiscence Therapy for People with Early-Stage of Alzheimer’s. Healthcare (Basel) 20210812th ed 2021 Aug 12;9(8). doi: 10.3390/healthcare9081036

90. Oliveira ALS, Matos LN, Colaco M, Delabrida ZNC. An Initial Assessment of a Chatbot for Rumination-Focused Cognitive Behavioral Therapy (RFCBT) in College Students. 2021. p. 549–564. doi: 10.1007/978-3-030-86979-3_39

91. Raji VPH, Maheswari PU. COVID-19 Lockdown in India: An Experimental Study on Promoting Mental Wellness Using a Chatbot during the Coronavirus. INTERNATIONAL JOURNAL OF MENTAL HEALTH PROMOTION 2022;24(2):189–205. doi: 10.32604/ijmhp.2022.011865

92. Rathnayaka P, Mills N, Burnett D, De Silva D, Alahakoon D, Gray R. A Mental Health Chatbot with Cognitive Skills for Personalised Behavioural Activation and Remote Health Monitoring. Sensors (Basel) 20220511th ed 2022 May 11;22(10). doi: 10.3390/s22103653

93. Roca S, Lozano ML, García J, Alesanco Á. Validation of a Virtual Assistant for Improving Medication Adherence in Patients with Comorbid Type 2 Diabetes Mellitus and Depressive Disorder. Int J Environ Res Public Health 20211117th ed 2021 Nov 17;18(22). doi: 10.3390/ijerph182212056

94. Sia DE, Yu MJ, Daliva JL, Montenegro J, Ong E, Sari E. Investigating the Acceptability and Perceived Effectiveness of a Chatbot in Helping Students Assess their Well-being. 2021. p. 34–40. doi: 10.1145/3429360.3468177

95. Sinha C, Cheng AL, Kadaba M. Adherence and Engagement With a Cognitive Behavioral Therapy-Based Conversational Agent (Wysa for Chronic Pain) Among Adults With Chronic Pain: Survival Analysis. JMIR Form Res 20220523rd ed 2022 May 23;6(5):e37302. doi: 10.2196/37302

96. Stephens TN. Behavioral intervention technologies for children and adolescents: Need, feasibility, and scalability. ProQuest Information & Learning; 2022. Available from: https://search.ebscohost.com/login.aspx?direct=true&db=psyh&AN=2022-13250-214&site=ehost-live

97. Tokunaga S, Tamura K, Otake-Matsuura M. A Dialogue-Based System with Photo and Storytelling for Older Adults: Toward Daily Cognitive Training. Front Robot AI 20210629th ed 2021;8:644964. doi: 10.3389/frobt.2021.644964

98. Trappey AJC, Lin APC, Hsu KYK, Trappey CV, Tu KLK. Development of an Empathy-Centric Counseling Chatbot System Capable of Sentimental Dialogue Analysis. PROCESSES 2022 May;10(5). doi: 10.3390/pr10050930

99. Williams R, Hopkins S, Frampton C, Holt-Quick C, Merry SN, Stasiak K. 21-Day Stress Detox: Open Trial of a Universal Well-Being Chatbot for Young Adults. SOCIAL SCIENCES-BASEL 2021 Nov;10(11). doi: 10.3390/socsci10110416

100. Wong J, Foussat AC, Ting S, Acerbi E, van Elburg RM, Mei Chien C. A Chatbot to Engage Parents of Preterm and Term Infants on Parental Stress, Parental Sleep, and Infant Feeding: Usability and Feasibility Study. JMIR Pediatr Parent 2021 Oct 26;4(4):e30169. PMID:34544679

101. Xin TL, Arshad A, Salam ZAB, Ieee. AlzBot- Mobile App Chatbot for Alzheimer’s Patient to be Active with Their Minds. 2021. p. 124–129. doi: 10.1109/DESE54285.2021.9719410

102. Suganuma S, Sakamoto D, Shimoyama H. An embodied conversational agent for unguided internet-based cognitive behavior therapy in preventative mental health: Feasibility and acceptability pilot trial. JMIR Ment Health 2018 Jul 31;5(3):e10454. PMID:30064969

103. 19th International Congress of the Pacific Rim College of Psychiatrists. Asia-pacific psychiatry 2021;13(SUPPL 1). Available from: https://www.cochranelibrary.com/central/doi/10.1002/central/CN-02263710/full

104. Bendig EE Benjamin; Meißner, Dominik; Bauereiß, Natalie; Baumeister, Harald. Feasibility of a Software agent providing a brief Intervention for Self-help to Uplift psychological wellbeing (“SISU”). A single-group pretest-posttest trial investigating the potential of SISU to act as therapeutic agent. Internet interventions 2021;24(NA):100377-NA. doi: 10.1016/j.invent.2021.100377

105. Ellis-Brush K. Augmenting Coaching Practice Through Digital Methods. International Journal of Evidence Based Coaching and Mentoring 2021;S15:187–197. doi: 10.24384/er2p-4857

106. Hunt M, Miguez S, Dukas B, Onwude O, White S. Efficacy of Zemedy, a mobile digital therapeutic for the self-management of irritable bowel syndrome: Crossover randomized controlled trial. JMIR Mhealth Uhealth 2021 May 20;9(5):e26152. PMID:33872182

107. Jang S, Kim J-J, Kim S-J, Hong J, Kim S, Kim E. Mobile app-based chatbot to deliver cognitive behavioral therapy and psychoeducation for adults with attention deficit: A development and feasibility/usability study. International Journal of Medical Informatics 2021 Jun;150:104440. PMID:33799055

108. Prochaska JJ; V Erin A; Chieng, Amy; Kendra, Matthew S; Baiocchi, Michael; Pajarito, Sarah; Robinson, Athena. A Therapeutic Relational Agent for Reducing Problematic Substance Use (Woebot): Development and Usability Study. Journal of medical Internet research 2021;23(3):e24850-NA. doi: 10.2196/24850

109. Troitskaya O, Batkhina A. Mobile application for couple relationships: Results of a pilot effectiveness study. Fam Process United States; 2022 Jun;61(2):625–642. PMID:34904235

110. Danieli MC Tommaso; Mousavi, Seyed Mahed; Silvestri, Giorgia; Barbato, Simone; Di Natale, Lorenzo; Riccardi, Giuseppe. Assessing the Impact of Conversational Artificial Intelligence in the Treatment of Stress and Anxiety in Aging Adults: Randomized Controlled Trial. JMIR mental health 2022;9(9):e38067–e38067. doi: 10.2196/38067

111. Selaskowski BS Maria; Schulze, Marcel; Lingen, Meike; Aslan, Behrem; Rosen, Helena; Kannen, Kyra; Wiebe, Annika; Wallbaum, Torben; Boll, Susanne; Lux, Silke; Philipsen, Alexandra; Braun, Niclas. Smartphone-assisted psychoeducation in adult attention-deficit/hyperactivity disorder: A randomized controlled trial. Psychiatry research 2022;317(NA):114802–114802. doi: 10.1016/j.psychres.2022.114802

112. Anderson PL; P Matthew; Edwards, Shannan M; Obasaju, Mayowa A; Schmertz, Stefan K; Zimand, Elana; Calamaras, Martha R. Virtual reality exposure therapy for social anxiety disorder: a randomized controlled trial. Journal of consulting and clinical psychology 2013;81(5):751–760. doi: 10.1037/a0033559

113. Fuhr K, Fahse B, Hautzinger M, Gulewitsch MD. Implementation of an Internet-Based Self-Help for Patients Waiting for Outpatient Psychotherapy - First Results. Psychother Psychosom Med Psychol 2018 Jun;68(6):234–241. PMID:29351710

114. Anguera JA; J Joshua T; Castaneda, Diego; Gazzaley, Adam; Areán, Patricia A. Conducting a fully mobile and randomised clinical trial for depression: access, engagement and expense. BMJ innovations 2016;2(1):14–21. doi: 10.1136/bmjinnov-2015-000098

115. Bentz DW Nan; Ibach, Merle K; Schicktanz, Nathalie; Zimmer, Anja; Papassotiropoulos, Andreas; de Quervain, Dominique JF. Effectiveness of a stand-alone, smartphone-based virtual reality exposure app to reduce fear of heights in real-life: a randomized trial. NPJ digital medicine 2021;4(1):16–16. doi: 10.1038/s41746-021-00387-7

116. Broglia EM Abigail; Barkham, Michael. Counseling With Guided Use of a Mobile Well-Being App for Students Experiencing Anxiety or Depression: Clinical Outcomes of a Feasibility Trial Embedded in a Student Counseling Service. JMIR mHealth and uHealth 2019;7(8):e14318-NA. doi: 10.2196/14318

117. Casey LM; O Tian PS; Raylu, Namrata; Horrigan, Katherine; Day, Jamin; Ireland, Michael J; Clough, Bonnie A. Internet-Based Delivery of Cognitive Behaviour Therapy Compared to Monitoring, Feedback and Support for Problem Gambling: A Randomised Controlled Trial. Journal of gambling studies 2017;33(3):993–1010. doi: 10.1007/s10899-016-9666-y

118. Dear BF; Z Judy; Ali, Shehzad; Lorian, Carolyn N; Johnston, Luke; Sheehan, Joanne; Staples, Lauren G; Gandy, Milena; Fogliati, Vincent J; Klein, Britt; Titov, Nickolai. Clinical and cost-effectiveness of therapist-guided internet-delivered cognitive behavior therapy for older adults with symptoms of anxiety: a randomized controlled trial. Behavior Therapy 2015;46(2):193–205. doi: 10.1016/j.beth.2014.09.008

119. Donker TC Ilja; van Klaveren, Chris; van Straten, Annemieke; Carlbring, Per; Cuijpers, Pim; van Gelder, Jean-Louis. Effectiveness of Self-guided App-Based Virtual Reality Cognitive Behavior Therapy for Acrophobia: A Randomized Clinical Trial. JAMA psychiatry 2019;76(7):682–690. doi: 10.1001/jamapsychiatry.2019.0219

120. Donkin LH Ian B; Christensen, Helen; Naismith, Sharon L; Neal, Bruce; Cockayne, Nicole; Glozier, Nick. Rethinking the dose-response relationship between usage and outcome in an online intervention for depression: randomized controlled trial. Journal of medical Internet research 2013;15(10):1–14. doi: 10.2196/jmir.2771

121. Gonzalez VM; D Patrick L. Comparison of a Smartphone App for Alcohol Use Disorders With an Internet-based Intervention Plus Bibliotherapy: A Pilot Study. Journal of consulting and clinical psychology 2015;83(2):335–345. doi: 10.1037/a0038620

122. Hester RK; S Daniel D; Delaney, Harold D. The Drinker’s Check-up: 12-month outcomes of a controlled clinical trial of a stand-alone software program for problem drinkers. Journal of substance abuse treatment 2005;28(2):159–169. doi: 10.1016/j.jsat.2004.12.002

123. Kocur MD Martin; Wolff, Christian; Nothdurfter, Caroline; Wetter, Thomas C; Rupprecht, Rainer; Shiban, Youssef. Computer-Assisted Avatar-Based Treatment for Dysfunctional Beliefs in Depressive Inpatients: A Pilot Study. Frontiers in psychiatry 2021;12(NA):608997-NA. doi: 10.3389/fpsyt.2021.608997

124. Mantani A, Kato T, Furukawa TA, Horikoshi M, Imai H, Hiroe T, Chino B, Funayama T, Yonemoto N, Zhou Q, Kawanishi N. Smartphone Cognitive Behavioral Therapy as an Adjunct to Pharmacotherapy for Refractory Depression: Randomized Controlled Trial. J Med Internet Res 2017 Nov 3;19(11):e373. doi: 10.2196/jmir.8602

125. McCall HC; R Chris G; Helgadottir, Fjola Dogg; Chen, Frances S. Evaluating a Web-Based Social Anxiety Intervention Among University Students: Randomized Controlled Trial. Journal of medical Internet research 2018;20(3):e91-NA. doi: 10.2196/jmir.8630

126. Premkumar PH Nadja; Brown, David; Battersby, Steven; Sumich, Alexander; Huntington, Bethany; Daly, Rosie C; Zysk, Eva. The Effectiveness of Self-Guided Virtual-Reality Exposure Therapy for Public-Speaking Anxiety. Frontiers in psychiatry 2021;12(NA):694610-NA. doi: 10.3389/fpsyt.2021.694610

127. Proudfoot J; R Clash; Everitt, Brian; Shapiro, David A; Goldberg, David; Mann, Anthony; Tylee, Andre; Marks, Isaac M; Gray, Jeffrey A. Clinical efficacy of computerised cognitive-behavioural therapy for anxiety and depression in primary care: randomised controlled trial. The British journal of psychiatry : the journal of mental science 2004;185(1):46–54. doi: 10.1192/bjp.185.1.46

128. Ritterband LM; T Frances P; Ingersoll, Karen S; Lord, Holly R; Gonder-Frederick, Linda; Frederick, Christina; Quigg, Mark; Cohn, Wendy F; Morin, Charles M. Effect of a Web-Based Cognitive Behavior Therapy for Insomnia Intervention With 1-Year Follow-up: A Randomized Clinical Trial. JAMA psychiatry 2017;74(1):68–75. doi: 10.1001/jamapsychiatry.2016.3249

129. Sun SL Danhua; Goldberg, Simon B; Shen, Zijiao; Chen, Pujing; Qiao, Shan; Brewer, Judson A; Loucks, Eric B; Operario, Don. A mindfulness-based mobile health (mHealth) intervention among psychologically distressed university students in quarantine during the COVID-19 pandemic: A randomized controlled trial. Journal of counseling psychology 2021;69(2):157–171. doi: 10.1037/cou0000568

130. Taylor CBB Susan W; Luce, Kristine H; Cunning, Darby; Doyle, Angela Celio; Abascal, Liana; Rockwell, Roxanne; Dev, Pavarti; Winzelberg, Andrew J; Wilfley, Denise E. Prevention of Eating Disorders in At-Risk College-Age Women. Archives of general psychiatry 2006;63(8):881–888. doi: 10.1001/archpsyc.63.8.881

131. Taylor CBK Andrea E; Trockel, Mickey; Cunning, Darby; Weisman, Hannah; Bailey, Jakki O; Sinton, Meghan M; Aspen, Vandana; Schecthman, Kenneth B; Jacobi, Corinna; Wilfley, Denise E. Reducing eating disorder onset in a very high risk sample with significant comorbid depression: A randomized controlled trial. Journal of consulting and clinical psychology 2016;84(5):402–414. doi: 10.1037/ccp0000077

132. Titov ND Blake F; Ali, Shehzad; Zou, Judy B; Lorian, Carolyn N; Johnston, Luke; Terides, Matthew D; Kayrouz, Rony; Klein, Britt; Gandy, Milena; Fogliati, Vincent J. Clinical and cost-effectiveness of therapist-guided internet-delivered cognitive behavior therapy for older adults with symptoms of depression: a randomized controlled trial. Behavior therapy 2014;46(2):193–217. doi: 10.1016/j.beth.2014.09.007

133. Titov ND Blake F; Johnston, Luke; Lorian, Carolyn N; Zou, Judy; Wootton, Bethany M; Spence, Jay; McEvoy, Peter M; Rapee, Ronald M. Improving Adherence and Clinical Outcomes in Self-Guided Internet Treatment for Anxiety and Depression: Randomised Controlled Trial. PloS one 2013;8(7):11-NA. doi: 10.1371/journal.pone.0062873

134. Warmerdam LS Filip; van Straten, Annemieke; Riper, Heleen; Cuijpers, Pim. Cost-utility and cost-effectiveness of internet-based treatment for adults with depressive symptoms: randomized trial. Journal of medical Internet research 2010;12(5):e53-NA. doi: 10.2196/jmir.1436

135. Merkouris SS; L G; Abdelrazek, M; Rodda, SN; Ibrahim, A; Bonti, A; Dowling, NA. Improving the user experience of a gambling support and education website using a chatbot. Universal Access in the Information Society 2022;NA(NA):NA-NA. doi: 10.1007/s10209-022-00932-5

136. Perski OC David; Beard, Emma; Brown, Jamie. Does the addition of a supportive chatbot promote user engagement with a smoking cessation app? An experimental study. Digital health 2019;5(NA):2055207619880676–2055207619880676. doi: 10.1177/2055207619880676

137. Gardiner PM, McCue KD, Negash LM, Cheng T, White LF, Yinusa-Nyahkoon L, Jack BW, Bickmore TW. Engaging women with an embodied conversational agent to deliver mindfulness and lifestyle recommendations: A feasibility randomized control trial. Patient Education and Counseling 2017;100(9):1720–1729. doi: 10.1016/j.pec.2017.04.015

138. Probst T, Berger T, Meyer B, Späth C, Schröder J, Hohagen F, Moritz S, Klein JP. Social phobia moderates the outcome in the EVIDENT study: A randomized controlled trial on an Internet-based psychological intervention for mild to moderate depressive symptoms. J Consult Clin Psychol 2020 Jan;88(1):82–89. PMID:31682137

139. Greer S, Ramo D, Chang Y-J, Fu M, Moskowitz J, Haritatos J. Use of the chatbot “Vivibot” to deliver positive psychology skills and promote well-being among young people after cancer treatment: Randomized controlled feasibility trial. JMIR Mhealth Uhealth 2019 Oct 31;7(10):e15018. PMID:31674920

140. Burton C, Szentagotai Tatar A, McKinstry B, Matheson C, Matu S, Moldovan R, Macnab M, Farrow E, David D, Pagliari C, Serrano Blanco A, Wolters M, for the Help4Mood Consortium. Pilot randomised controlled trial of Help4Mood, an embodied virtual agent-based system to support treatment of depression. J Telemed Telecare 2016 Sep;22(6):348–355. PMID:26453910

141. Fitzpatrick KK, Darcy A, Vierhile M. Delivering cognitive behavior therapy to young adults with symptoms of depression and anxiety using a fully automated conversational agent (Woebot): A randomized controlled trial. JMIR Ment Health 2017 Jun 6;4(2):e19. PMID:28588005

142. Fulmer R, Joerin A, Gentile B, Lakerink L, Rauws M. Using psychological artificial intelligence (Tess) to relieve symptoms of depression and anxiety: Randomized controlled trial. JMIR Ment Health 2018 Dec 13;5(4):e64. PMID:30545815

143. Liu H, Peng H, Song X, Xu C, Zhang M. Using AI chatbots to provide self-help depression interventions for university students: A randomized trial of effectiveness. Internet Interv 20220106th ed 2022 Mar;27:100495. doi: 10.1016/j.invent.2022.100495

144. Narain J, Quach T, Davey M, Park HW, Breazeal C, Picard R, Assoc Comp M. Promoting Wellbeing with Sunny, a Chatbot that Facilitates Positive Messages within Social Groups. 2020. doi: 10.1145/3334480.3383062

145. Oh J, Jang S, Kim H, Kim J-J. Efficacy of mobile app-based interactive cognitive behavioral therapy using a chatbot for panic disorder. International Journal of Medical Informatics 2020 Aug;140:104171. PMID:32446158

146. Ly KHL Ann-Marie; Andersson, Gerhard. A fully automated conversational agent for promoting mental well-being: A pilot RCT using mixed methods. Internet interventions 2017;10(NA):39–46. doi: 10.1016/j.invent.2017.10.002

147. Danieli MC Tommaso; Mousavi, Seyed Mahed; Riccardi, Giuseppe. A Conversational Artificial Intelligence Agent for a Mental Health Care App: Evaluation Study of Its Participatory Design. JMIR formative research 2021;5(12):e30053-NA. doi: 10.2196/30053

148. Shamekhi A, Bickmore TW, Lestoquoy A, Gardiner P. Augmenting Group Medical Visits with Conversational Agents for Stress Management Behavior Change. 2017. p. 55–67. doi: 10.1007/978-3-319-55134-0_5

149. So R, Furukawa TA, Matsushita S, Baba T, Matsuzaki T, Furuno S, Okada H, Higuchi S. Unguided chatbot-delivered cognitive behavioural intervention for problem gamblers through messaging app: A randomised controlled trial. J Gambl Stud 2020 Dec;36(4):1391–1407. PMID:32162075

150. Klos MC, Escoredo M, Joerin A, Lemos VN, Rauws M, Bunge EL. Artificial Intelligence-Based Chatbot for Anxiety and Depression in University Students: Pilot Randomized Controlled Trial. JMIR Form Res 2021 Aug 12;5(8):e20678. PMID:34092548
