# Supplementary figures and images for: Attrition in Conversational Agent–Delivered Mental Health Interventions: Systematic Review and Meta-Analysis
Source: J Med Internet Res. 2024 Feb 27;26:e48168. doi: 10.2196/48168 (PMC10933752; doi:10.2196/48168)

# Multimedia Appendix 5: Risk of bias assessment of the included studies


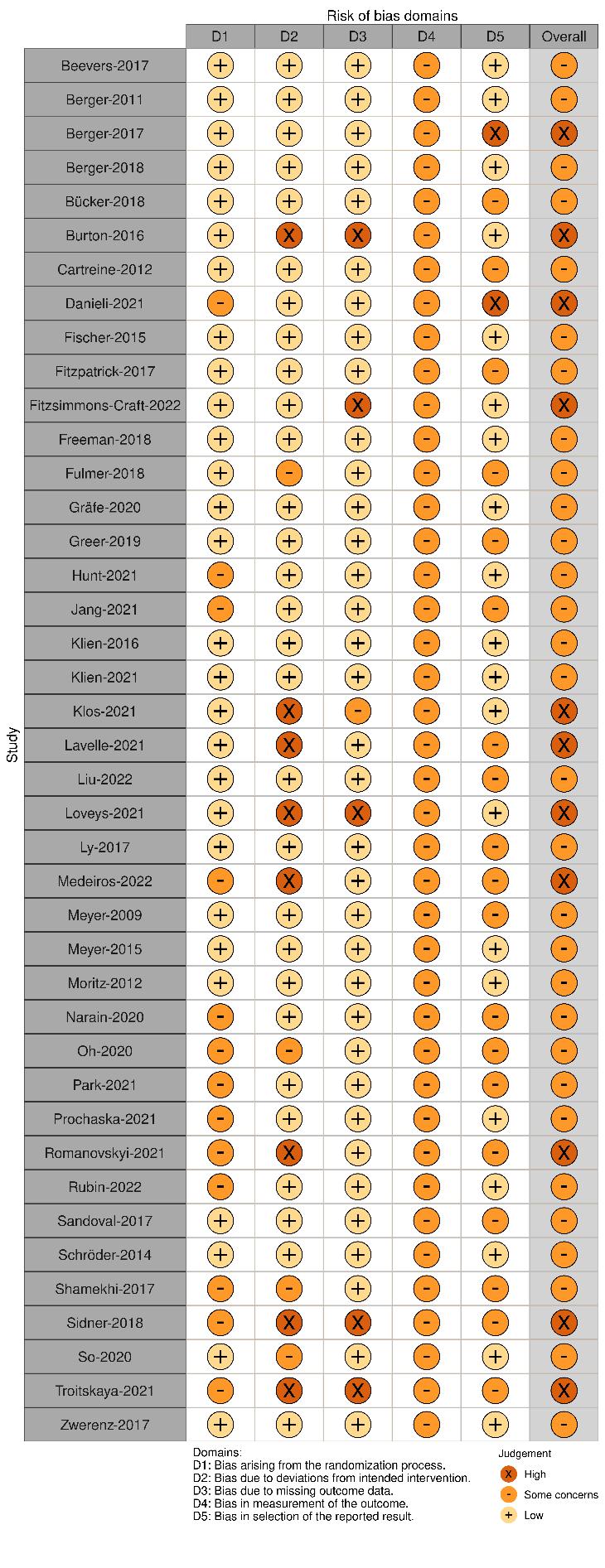

Supplement: Multimedia Appendix 5 [file jmir_v26i1e48168_app5.docx]
