# Supplementary material for: Attrition in Conversational Agent–Delivered Mental Health Interventions: Systematic Review and Meta-Analysis
Source: J Med Internet Res. 2024 Feb 27;26:e48168. doi: 10.2196/48168 (PMC10933752; doi:10.2196/48168)

| Multimedia Appendix 6: Differential attrition analysis for short- and long-term studies. Table 1: Sub-group analysis of the differential attrition for short- and long-term studies. | | | | | | | | | |
| --- | --- | --- | --- | --- | --- | --- | --- | --- | --- |
|  |  | Short-term (≤ 8 weeks) | | | | Long-term (> 8 weeks) | | | |
| Subgroups | | *k* | Event rate, log OR [95% CI] | *I2* (%) | *p* | *k* | Attrition rate, % [95% CI] | *I2* (%) | *p* |
| Risk of Bias | |  |  |  | 0.81 |  |  |  | 0.958 |
|  | High Risk of Bias | 7 | 1.23 [0.93; 1.63] | 0 |  | 4 | 1.2132 [0.8039; 1.8310] | 27.7 |  |
|  | Low Risk of Bias | 12 | 1.30 [0.95; 1.77] | 12.5 |  | 10 | 1.2279 [1.0293; 1.4647] | 30.7 |  |
| Funding source | |  |  |  | 0.07 |  |  |  | 0.965 |
|  | Industry funding | 8 | 1.6637 [1.1694; 2.3669] | 0 |  | 4 | 1.2348 [0.9819; 1.5529] | 36.3 |  |
|  | Public funding only | 11 | 1.1179 [0.8801; 1.4199] | 0 |  | 10 | 1.2251 [0.9418; 1.5937] | 27.2 |  |
| Durations | |  |  |  | **0.016** |  |  |  | 0.4218 |
|  | 0-4 weeks | 17 | 0.9931 [0.7502; 1.3147] | 0 |  |  |  |  |  |
|  | 5-8 weeks | 9 | 1.6134 [1.2202; 2.1334] | 0 |  |  |  |  |  |
|  | 9-12 weeks |  |  |  |  | 10 | 1.1206 [0.9945; 1.2625] | 0 |  |
|  | >13 weeks |  |  |  |  | 4 | 1.3364 [0.8852; 2.0176] | 42.3 |  |
| Study Design | |  |  |  | 0.304 |  |  |  | 0.206 |
|  | RCT | 14 | 1.3272 [1.0583; 1.6644] | 5.2 |  | 13 | 1.2008 [1.0324; 1.3966] | 22 |  |
|  | Pilot RCT | 5 | 0.9945 [0.6023; 1.6422] | 0 |  | 1 | 2.3333 [0.8438; 6.4526] |  |  |
| Type of Disorders | |  |  |  | 0.162 |  |  |  | **0.003** |
|  | Depression | 5 | 1.6196 [1.1141; 2.3546] | 16.5 |  | 10 | 1.0853 [0.9624; 1.2238] | 0 |  |
|  | Depression and Anxiety | 2 | 0.8374 [0.2305; 3.0420] | 22.5 |  | - |  |  |  |
|  | Mental Well-being | 6 | 0.9615 [0.6787; 1.3620] | 0 |  | - |  |  |  |
|  | Others | 6 | 1.4842 [1.0232; 2.1529] | 0 |  | 4 | 1.6278 [1.2763; 2.0762] | 0 |  |
| With CBT | |  |  |  |  |  |  |  | 0.198 |
|  | CBT | 13 | 1.2669 [1.0003; 1.6045] | 18.5 | 0.945 | 11 | 1.1749 [1.0122; 1.3638] | 22.8 |  |
|  | No CBT | 6 | 1.2432 [0.7672; 2.0147] | 0 |  | 3 | 1.5766 [1.0336; 2.4049] | 9.3 |  |
| With Mindfulness | |  |  |  | 0.671 |  |  |  | **0.025** |
|  | Mindfulness | 11 | 1.3049 [1.0323; 1.6496] | 19.1 |  | 10 | 1.1059 [0.9807; 1.2470] | 0 |  |
|  | No Mindfulness | 8 | 1.1778 [0.8139; 1.7043] | 0 |  | 4 | 1.5067 [1.1814; 1.9214] | 22.8 |  |
| Personalization | |  |  |  | 0.179 |  |  |  | 0.071 |
|  | No personalization | 5 | 1.7804 [0.9978; 3.1767] | 0 |  | 2 | 1.5170 [1.1511; 1.9992] | 0 |  |
|  | Minimal personalization | 2 | 0.8210 [0.4570; 1.4751] | 0 |  |  |  |  |  |
|  | Substantial personalization | 7 | 1.4137 [1.0651; 1.8764] | 12.3 |  | 11 | 1.1316 [1.0059; 1.2730] | 15.7 |  |
|  | Major personalization | 5 | 1.0460 [0.7029; 1.5565] | 2 |  | 1 | 0.6061 [0.2181; 1.6843] | 0 |  |
| CA Algorithm | |  |  |  | 0.816 |  |  |  |  |
|  | Rule-based | 12 | 1.2811 [0.9693; 1.6933] | 16 |  | 12 | 1.1406 [1.0154; 1.2812] | 12.3 |  |
|  | AI-enhanced | 7 | 1.2145 [0.8546; 1.7260] | 0 |  | 2 | 1.0967 [0.4665; 2.5783] | 64.7 |  |
| Type of CA | |  |  |  | 0.241 |  |  |  | 0.393 |
|  | No avatar | 3 | 1.7811 [1.2125; 2.6162] | 0 |  | 11 | 1.2477 [1.0524; 1.4792] |  |  |
|  | ECA | 5 | 1.3470 [0.3593; 5.0490] | 28.7 |  | 2 | 1.2417 [0.6729; 2.2913] |  |  |
|  | Avatar | 9 | 1.1647 [0.8818; 1.5382] | 0 |  | 1 | 0.6061 [0.2181; 1.6843] |  |  |
|  | Not specified | 2 | 0.9604 [0.5335; 1.7290] | 37.9 |  |  |  |  |  |
| Rewards | |  |  |  | 0.845 |  |  |  | 0.076 |
|  | With rewards | 11 | 1.2457 [0.9634; 1.6108] | 0 |  | 1 | 1.5114 [1.1220; 2.0360] | 14.3 |  |
|  | No rewards | 8 | 1.3044 [0.8888; 1.9144] | 23.6 |  | 13 | 1.1314 [1.0080; 1.2699] | 0 |  |
| Reminder | |  |  |  | 0.430 |  |  |  | 0.119 |
|  | With reminder | 12 | 1.1812 [0.8960; 1.5572] | 0 |  | 7 | 1.3494 [1.0761; 1.6922] | 33.8 |  |
|  | Without reminder | 7 | 1.3926 [1.0301; 1.8829] | 17.9 |  | 7 | 1.0944 [0.9567; 1.2520] | 0 |  |
| Delivery Channel | |  |  |  |  |  |  |  | 0.220 |
|  | Web-based | 4 | 1.8117 [1.2394; 2.6482] | 0 | 0.146 | 10 | 1.1366 [1.0096; 1.2796] | 21.2 |  |
|  | Computer-based | 4 | 1.0644 [0.2217; 5.1090] | 36.3 |  | 1 | 0.8007 [0.2823; 2.2711] | 0 |  |
|  | Smartphone app | 6 | 1.2389 [0.8312; 1.8465] | 10.3 |  | 1 | 1.5114 [1.1220; 2.0360] | 0 |  |
|  | Messenger-based | 5 | 1.0018 [0.7187; 1.3965] | 0 |  | 1 | 0.6061 [0.2181; 1.6843] | 0 |  |
|  | Not specified |  |  |  |  | 1 | 1.5514 [0.7451; 3.2299] | 0 |  |
| Blended Design | |  |  |  | 0.780 |  |  |  | 0.120 |
|  | with Blended | 2 | 0.9081 [0.0827; 9.9688] | 60.5 |  | 6 | 1.0575 [0.8382; 1.3342] | 0 |  |
|  | Without | 17 | 1.2794 [1.0477; 1.5623] | 0 |  | 9 | 1.3871 [1.0795; 1.7825] | 44.1 |  |
| Enrolment method | |  |  |  | 0.664 |  |  |  | 0.207 |
|  | Remote options only | 11 | 1.3134 [1.0445; 1.6515] | 15 |  | 9 | 1.3613 [1.0449; 1.7735] | 52.9 |  |
|  | With inperson option | 7 | 1.1131 [0.6717; 1.8446] | 0 |  | 6 | 1.0866 [0.8643; 1.3661] | 0 |  |
|  | Not specified | 1 | 0.7579 [0.1753; 3.2776] | 0 |  |  |  |  |  |
| Study Population | |  |  |  | **0.009** |  |  |  | 0.993 |
|  | At-risk | 9 | 1.6478 [1.2608; 2.1535] |  |  | 6 | 1.2340 [0.9547; 1.5949] | 21.8 |  |
|  | Clinical | 3 | 0.4654 [0.1302; 1.6635] |  |  | 8 | 1.2320 [0.9759; 1.5553] | 31 |  |
|  | General | 7 | 0.9591 [0.7090; 1.2975] |  |  |  |  |  |  |
| Type of comparison | |  |  |  | **0.030** |  |  |  | 0.201 |
|  | Wait-list | 11 | 1.5193 [1.1834; 1.9505] |  |  | 4 | 1.4637 [1.1161; 1.9195] | 28.9 |  |
|  | Treatment as usual | 0 |  |  |  | 7 | 1.1147 [0.9856; 1.2607] | 0 |  |
|  | Active control | 7 | 0.9596 [0.6880; 1.3385] |  |  | 3 | 1.1765 [0.6296; 2.1986] | 57 |  |
| Session Length | |  |  |  | 0.400 |  |  |  | 0.174 |
|  | Defined session length | 11 | 1.3544 [1.0216; 1.7957] |  |  | 13 | 1.2424 [1.0599; 1.4563] | 22.7 |  |
|  | User determined | 8 | 1.1195 [0.7951; 1.5763] |  |  | 1 | 0.6061 [0.2181; 1.6843] | 0 |  |
| Symptom Trackers | |  |  |  | 0.1633 |  |  |  | 0.402 |
|  | With Symptom Trackers | 10 | 1.0824 [0.8114; 1.4439] |  |  | 6 | 1.0116 [0.6197; 1.6512] | 32 |  |
|  | Without Symptom Trackers | 9 | 1.4650 [1.0711; 2.0036] |  |  | 8 | 1.2640 [1.0587; 1.5092] | 25.7 |  |

Figure 1: Forest plot for differential meta-analysis of attrition rate of the included studies.
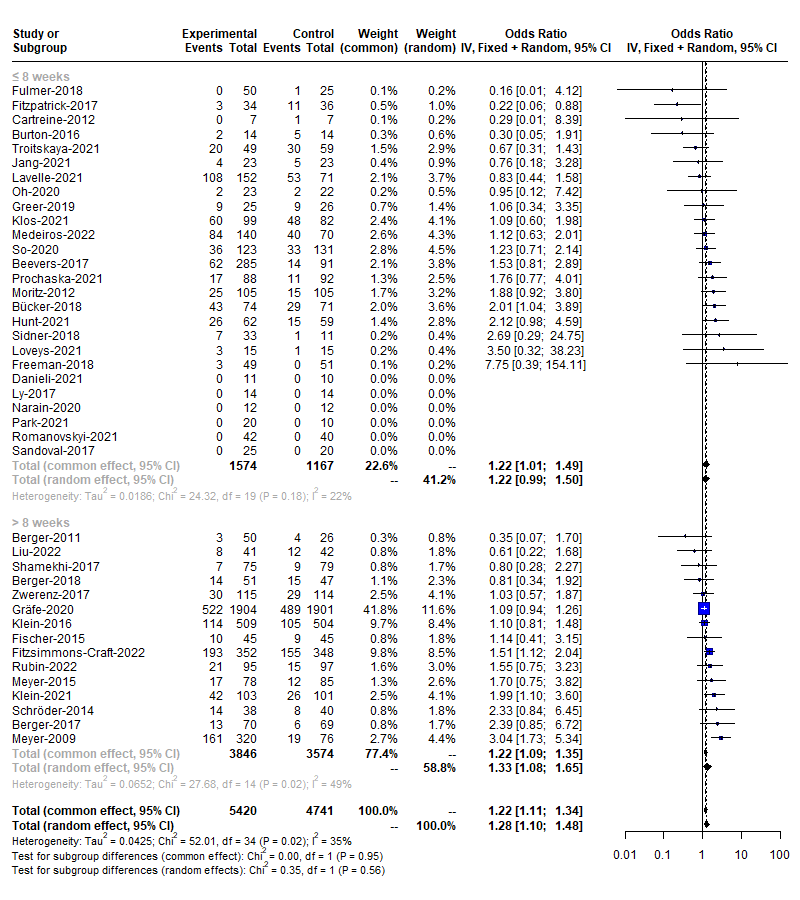

Supplement: Multimedia Appendix 6 [file jmir_v26i1e48168_app6.docx]
