# Supplementary material for: Attrition in Conversational Agent–Delivered Mental Health Interventions: Systematic Review and Meta-Analysis
Source: J Med Internet Res. 2024 Feb 27;26:e48168. doi: 10.2196/48168 (PMC10933752; doi:10.2196/48168)
Supplement: Multimedia Appendix 7 [file jmir_v26i1e48168_app7.docx]

# Multimedia Appendix 7: Extended result for the exploratory sub-group analysis.

Exploratory sub-group analyses using the pre-specified subgroup analyses were conducted to explain the heterogeneity of the included studies regardless of the duration of the interventions. For the overall attrition, like our prespecified analysis, there were significant differences in the attrition rates in the intervention group depending on the inclusion of mindfulness content, χ^2^ (1) = 3.99, p=0.05. However, we did not find significant differences in the inclusion of blended support compared to non-blended intervention. Additionally, we also found significant differences depending on the type of CA used, χ^2^ (3) = 13.06, p=.005; the CA delivery channel, χ^2^ (4) = 21.27, p < .001, and study enrolment method, χ^2^ (2) = 7.44, p=.02. Studies that included mindfulness content (k = 23) reported higher rate of attrition in the intervention group 27.32 (95% CI [20.98; 34.15]), compared to those without mindfulness content, k = 18, 13.94 (95% CI [5.54; 24.86]). Studies that did not use any identifiable avatar reported the highest rate of attrition, k = 15, 30.0% (95% CI [23.44; 37.01], followed by studies that did not specify their use of avatar or visual representation of the CA, k = 14, 20.12% (95% CI [7.29; 36.82], static avatar, k = 4, 15.15% (95% CI [1.79; 35.93]), and ECA, k = 8, 10.30% (95% CI [ 4.29; 18.04]). Sensitivity analysis conducted using only sub-group with 5 or more studies was still statistically significant, χ^2^ (2) = 12.6, p=.002. Interventions that were delivered via messenger-based intervention showed the highest rate of attrition, k = 7, 31.19% (95% CI [10.68; 56.28]), followed by web-based intervention, k = 15, 27.90% (95% CI [22.35; 33.78]), and standalone smartphone apps, k = 11, 17.36% (95% CI [ 6.54; 31.48]. CA installed on computer, laptop, or tablet showed the lowest rate of attrition, k = 7, 5.61% (95% CI [1.09; 12.30]). One study did not specify the delivery channel used [86]. Lastly, studies that offered remote onboarding only (k = 23) showed the higher attrition rate, 28.42% (95% CI [21.30; 36.10]) compared to studies that offered in-person onboarding process, k = 16, 15.01% (95% CI [ 8.46; 22.82]). The sub-group analysis was still significantly different after dropping the two studies [75,81] that did not report their onboarding process (Table 1).

For differential attrition, our findings were mostly similar to our pre-specified analysis. There were significant differences depending on the duration of the intervention, χ^2^ (3) = 7.78, *p* = .05; type of disorder, χ^2^ (3) = 8.53, *p* = .04; study population, χ^2^ (2) = 8.43, *p* = .01; type of comparison group, χ^2^ (2) = 10.58, *p* = .005. Unlike the pre-specified analysis, the was a significant difference for studies that included symptom trackers, χ^2^ (1) = 5.04, *p* = .02. The risk of attrition in the intervention group was lower compared to control group for studies that lasted between 0 to 4 weeks, k = 7, log OR = 0.94 (95% CI [0.71; 1.23]) and studies that between 5 to 8 weeks, k = 12, log OR = 0.99 (95% CI [0.75, 1.31]). The risk of attrition in the intervention group was higher compared to control group for studies that lasted between 9 to 12 weeks, k = 11, log OR = 1.34 (95% CI [1.02; 1.75]), and studies that lasted more than 13 weeks, k = 4, log OR = 1.45 (95% CI [1.13; 1.86]). Studies that targeted mental well-being specifically (k = 6) showed relatively lower attrition rate in the intervention group compared to the control groups, log OR = 0.96 (95% CI [0.68; 1.36]), relative to studies that targeted depression symptoms only, k = 16, log OR = 1.27 (95% CI [1.01; 1.61]), and studies that targeted other mental health conditions such as phobia and substance abuse, k = 10, log OR = 1.58 (95% CI [1.29; 1.94]). The sub-group analysis was still significantly different after dropping 3 studies that focused on both depression and anxiety, χ^2^ (3) = 6.26, *p* = .04. Studies that recruited at-risk populations (k = 16) showed significantly higher attrition rates in the intervention group compared to control group participants, log OR = 1.51 (95% CI [1.23; 1.86]), when compared to general populations, k = 8, log OR = 0.90 (95% CI [0.67; 1.20], and clinical populations, k = 11, log OR = 1.18 (95% CI [0.96; 1.45]). The subgroup analysis was still significant when compared between general population and at-risk group only, χ^2^ (1) = 6.26, *p* = 0.04. Studies that employed wait-list control showed relatively higher attrition in the intervention group compared to control group, k = 16, log OR = 1.6109 (95% CI [1.30; 2.00]), compared to treatment-as-usual control group, k = 8, log OR = 1.11 (95% CI [0.9802; 1.2532]), and active control, k = 11, log OR = 0.97 (95% CI [0.72; 1.32]). Lastly, studies that included symptoms tracker (k =17) showed relatively lower attrition in the intervention group compared to control group, log OR = 1.02 (95% CI [0.81; 1.29]), compared to studies without symptoms tracker, k = 18, log OR = 1.44 (95% CI [1.19; 1.74]) (Table 2).

| Table 1: Sub-group analysis of overall attrition in the intervention group | | | | | |
| --- | --- | --- | --- | --- | --- |
| Subgroups | | *k* | Attrition rate, % [95% CI] | *I2* (%) | *p* |
| Risk of Bias | |  |  |  | 0.4081 |
|  | High Risk of Bias | 12 | 24.8826 [17.8538; 32.6023] | 84.9 |  |
|  | Low Risk of Bias | 29 | 20.5564 [14.1799; 27.6995] | 95.4 |  |
| Funding source | |  |  |  | 0.705 |
|  | Industry funding | 13 | 20.43 [8.78; 35.06] | 94.6 |  |
|  | Public funding only | 28 | 16.56 [6.35; 29.79] | 94.2 |  |
| Durations | |  |  |  | 0.6369 |
|  | 0-4 weeks | 17 | 15.6658 [ 5.1019; 29.9158] | 95.3 |  |
|  | 5-8 weeks | 9 | 23.1547 [11.1251; 37.6730] | 93.4 |  |
|  | 9-12 weeks | 11 | 25.3038 [19.3489; 31.7475] | 90.3 |  |
|  | >13 weeks | 4 | 30.0363 [10.7481; 53.8785] | 96.1 |  |
| Study Design |  |  |  |  | 0.5811 |
|  | RCT | 33 | 22.7299 [17.2955; 28.6412] | 94.7 |  |
|  | Pilot RCT | 8 | 15.9193 [ 1.9599; 37.0917] | 90.4 |  |
| Type of Disorders | |  |  |  | 0.7472 |
|  | Depression | 17 | 22.7009 [17.1677; 28.7227] | 91 |  |
|  | Depression and Anxiety | 5 | 7.6762 [ 0.0000; 40.9276] | 97.1 |  |
|  | Mental Well-being | 9 | 24.2890 [ 8.3667; 44.5677] | 94.2 |  |
|  | Others | 10 | 25.3859 [15.1388; 37.1646] | 92.9 |  |
| With CBT |  |  |  |  | 0.2193 |
|  | CBT | 29 | 24.4180 [18.5224; 30.8110] | 94.7 |  |
|  | No CBT | 12 | 14.8260 [ 4.6812; 28.5741] | 92.9 |  |
| With Mindfulness | |  |  |  | **0.0457** |
|  | Mindfulness | 23 | 27.3245 [20.9778; 34.1474] | 94.3 |  |
|  | No Mindfulness | 18 | 13.9449 [ 5.5447; 24.8571] | 94.3 |  |
| Personalization | |  |  |  | 0.595 |
|  | No personalization | 8 | 22.2026 [ 8.7838; 39.1767] | 93.7 |  |
|  | Minimal personalization | 2 | 44.5740 [ 2.4982; 92.4982] | 93.7 |  |
|  | Substantial personalization | 21 | 24.0689 [19.1911; 29.2915] | 90 |  |
|  | Major personalization | 10 | 11.8424 [ 0.1952; 33.2385] | 96.1 |  |
| CA Algorithm | |  |  |  | 0.4047 |
|  | Rule-based | 29 | 23.9909 [18.8041; 29.5652] | 92 |  |
|  | AI-enhanced | 12 | 16.9152 [ 4.8892; 33.5101] | 96.5 |  |
| Type of CA | |  |  |  | **0.0045** |
|  | No avatar | 15 | 30.0076 [23.4380; 37.0064] | 94.2 |  |
|  | ECA | 8 | 10.2991 [ 4.2905; 18.0391] | 65.1 |  |
|  | Avatar | 4 | 15.1479 [ 1.7893; 35.9261] | 86.4 |  |
|  | Not specified | 14 | 20.1158 [ 7.2913; 36.8172] | 96.2 |  |
| Rewards | |  |  |  | 0.5077 |
|  | With rewards | 16 | 18.8296 [ 9.4603; 30.2231] | 94.7 |  |
|  | No rewards | 25 | 23.4116 [17.3015; 30.0971] | 94 |  |
| Reminder | |  |  |  | 0.2621 |
|  | With reminder | 21 | 25.0121 [16.8813; 34.0822] | 94.8 |  |
|  | Without reminder | 20 | 18.3037 [11.5909; 26.0302] | 93.6 |  |
| Delivery Channel | |  |  |  | **0.0003** |
|  | Web-based | 15 | 27.8959 [22.3549; 33.7843] | 90.3 |  |
|  | Computer-based | 7 | 5.6057 [ 1.0920; 12.3026] | 55.1 |  |
|  | Smartphone app | 11 | 17.3555 [ 6.5381; 31.4805] | 94.3 |  |
|  | Messenger-based | 7 | 31.1865 [10.6779; 56.2780] | 96.7 |  |
|  | Not specified | 1 | 22.1053 [14.2737; 31.0559] | 0 |  |
| Blended Design | |  |  |  | 0.1498 |
|  | with Blended | 9 | 16.8566 [11.5089; 22.9026] | 68.5 |  |
|  | Without | 32 | 23.4134 [17.2098; 30.2055] | 94.9 |  |
| Enrolment method | |  |  |  | **0.0243** |
|  | Remote options only | 23 | 28.4228 [21.2999; 36.1048] | 95.3 |  |
|  | With inperson option | 16 | 15.0097 [ 8.4599; 22.8224] | 87.8 |  |
|  | Not specified | 2 | 5.0193 [ 0.0000; 33.0834] | 88 |  |
| Study Population | |  |  |  | 0.6131 |
|  | At-risk | 18 | 23.8066 [16.1867; 32.3275] | 94.4 |  |
|  | Clinical | 12 | 18.5333 [12.9838; 24.7433] | 81.2 |  |
|  | General | 11 | 22.0537 [ 6.7337; 42.2975] | 96.1 |  |
| Session Length | |  |  |  | 0.3074 |
|  | Defined session length | 29 | 24.4177 [18.7813; 30.5024] | 94 |  |
|  | User determined | 12 | 15.6885 [ 4.1169; 31.9951] | 95 |  |
| Symptom Trackers | |  |  |  | 0.2269 |
|  | With Symptom Trackers | 20 | 17.5086 [ 8.9722; 27.8842] | 93 |  |
|  | Without Symptom Trackers | 21 | 25.4039 [18.7588; 32.6482] | 95.2 |  |

| Table 2: Sub-group analysis of differential attrition between the intervention and control group | | | | | |
| --- | --- | --- | --- | --- | --- |
| Subgroups | | *k* | Event rate, log OR [95% CI] | *I2* (%) | *p* |
| Risk of Bias | |  |  |  | 0.3686 |
|  | High Risk of Bias | 12 | 1.1695 [0.9335; 1.4652] | 29.3 |  |
|  | Low Risk of Bias | 23 | 1.3383 [1.1082; 1.6162] | 39.4 |  |
| Funding source | |  |  |  | 0.7962 |
|  | Industry funding | 13 | 1.2978 [1.0566; 1.5942] | 39.6 |  |
|  | Public funding only | 22 | 1.2482 [1.0095; 1.5434] | 34.3 |  |
| Durations | |  |  |  | **0.0508** |
|  | 0-4 weeks | 13 | 0.9356 [0.7107; 1.2315] | 8.3 |  |
|  | 5-8 weeks | 7 | 1.6134 [1.2202; 2.1334] | 0 |  |
|  | 9-12 weeks | 11 | 1.3394 [1.0231; 1.7536] | 50.5 |  |
|  | >13 weeks | 4 | 1.4479 [1.1274; 1.8595] | 42.3 |  |
| Study Design | |  |  |  | 0.7078 |
|  | RCT | 29 | 1.2871 [1.1001; 1.5058] | 39.6 |  |
|  | Pilot RCT | 6 | 1.1750 [0.7493; 1.8425] | 11.7 |  |
| Type of Disorders | |  |  |  | **0.0362** |
|  | Depression | 16 | 1.2704 [1.0054; 1.6052] | 27.56 |  |
|  | Depression and Anxiety | 3 | 0.4810 [0.1268; 1.8251] | 5.33 |  |
|  | Mental Well-being | 6 | 0.9615 [0.6787; 1.3620] | 5.33 |  |
|  | Others | 10 | 1.5834 [1.2917; 1.9409] | 4.98 |  |
| With CBT | |  |  |  | 0.4825 |
|  | CBT | 26 | 1.1992 [1.0879; 1.3218] | 44.1 |  |
|  | No CBT | 9 | 1.4223 [1.0351; 1.9545] | 0 |  |
| With Mindfulness | |  |  |  | 0.8865 |
|  | Mindfulness | 22 | 1.2860 [1.0670; 1.5500] | 36.9 |  |
|  | No Mindfulness | 13 | 1.3139 [1.0470; 1.6488] | 31.5 |  |
| Personalization | |  |  |  | 0.0783 |
|  | No personalization | 7 | 1.5626 [1.2180; 2.0048] | 0.6 |  |
|  | Minimal personalization | 2 | 0.8210 [0.4570; 1.4751] | 0 |  |
|  | Substantial personalization | 20 | 1.3159 [1.0718; 1.6156] | 49 |  |
|  | Major personalization | 6 | 0.9736 [0.6722; 1.4102] | 0 |  |
| CA Algorithm | |  |  |  | 0.8646 |
|  | Rule-based | 26 | 1.2849 [1.0671; 1.5471] | 41.8 |  |
|  | AI-enhanced | 9 | 1.3183 [1.0476; 1.6591] | 4 |  |
| Type of CA | |  |  |  | 0.2152 |
|  | No avatar | 15 | 1.4492 [1.1858; 1.7712] | 51.1 |  |
|  | ECA | 7 | 1.2457 [0.7380; 2.1028] | 9.7 |  |
|  | Avatar | 2 | 0.9604 [0.5335; 1.7290] | 37.9 |  |
|  | Not specified | 11 | 1.0437 [0.7914; 1.3763] | 21.7 |  |
| Rewards | |  |  |  | 0.8632 |
|  | With rewards | 13 | 1.2736 [1.0249; 1.5825] | 27.3 |  |
|  | No rewards | 22 | 1.3069 [1.0715; 1.5942] | 39.7 |  |
| Reminder | |  |  |  | 0.6651 |
|  | With reminder | 20 | 1.2456 [1.0438; 1.4863] | 27.3 |  |
|  | Without reminder | 15 | 1.3334 [1.0356; 1.7168] | 45.3 |  |
| Delivery Channel | |  |  |  | 0.2059 |
|  | Web-based | 15 | 1.4632 [1.1656; 1.8367] | 49.4 |  |
|  | Computer-based | 5 | 0.8650 [0.3937; 1.9005] | 15.9 |  |
|  | Smartphone app | 8 | 1.1731 [0.8064; 1.7067] | 46 |  |
|  | Messenger-based | 6 | 0.9549 [0.6963; 1.3096] | 0 |  |
|  | Not specified | 1 | 1.5514 [0.7451; 3.2299] | 0 |  |
| Blended Design | |  |  |  | 0.0767 |
|  | with Blended | 8 | 1.0488 [0.8337; 1.3194] | 0 |  |
|  | Without | 27 | 1.3620 [1.1420; 1.6243] | 40.5 |  |
| Enrolment method | |  |  |  | 0.2515 |
|  | Remote options only | 21 | 1.3674 [1.1198; 1.6698] | 53 |  |
|  | With inperson option | 13 | 1.0910 [0.8857; 1.3440] | 0 |  |
|  | Not specified | 1 | 0.7579 [0.1753; 3.2776] | 0 |  |
| Study Population | |  |  |  | **0.0147** |
|  | At-risk | 16 | 1.5152 [1.2315; 1.8642] | 26.2 |  |
|  | Clinical | 11 | 1.1814 [0.9625; 1.4501] | 21.8 |  |
|  | General | 8 | 0.8967 [0.6675; 1.2046] | 12.6 |  |
| Type of comparison | |  |  |  | **0.0051** |
|  | Wait-list | 16 | 1.6109 [1.2997; 1.9967] | 18.4 |  |
|  | Treatment as usual | 8 | 1.1083 [0.9802; 1.2532] | 0 |  |
|  | Active control | 11 | 0.9730 [0.7153; 1.3236] | 24.9 |  |
| Session Length | |  |  |  | 0.063 |
|  | Defined session length | 25 | 1.3603 [1.1538; 1.6038] | 37.7 |  |
|  | User determined | 10 | 0.9585 [0.6889; 1.3337] | 20.4 |  |
| Symptom Trackers | |  |  |  | **0.0248** |
|  | With Symptom Trackers | 17 | 1.0212 [0.8100; 1.2875] | 10 |  |
|  | Without Symptom Trackers | 18 | 1.4383 [1.1905; 1.7377] | 46.2 |  |
